# A live dengue virus vaccine carrying a chimeric envelope glycoprotein elicits dual DENV2-DENV4 serotype-specific immunity

**DOI:** 10.1038/s41467-023-36702-x

**Published:** 2023-03-13

**Authors:** Ellen Young, Boyd Yount, Petraleigh Pantoja, Sandra Henein, Rita M. Meganck, Jennifer McBride, Jennifer E. Munt, Thomas J. Baric, Deanna Zhu, Trevor Scobey, Stephanie Dong, Longping V. Tse, Melween I. Martinez, Armando G. Burgos, Rachel L. Graham, Laura White, Aravinda DeSilva, Carlos A. Sariol, Ralph S. Baric

**Affiliations:** 1 Department of Epidemiology, Gillings School of Global Public Health, University of North Carolina, Chapel Hill, NC USA; 2 Unit of Comparative Medicine, University of Puerto Rico-Medical Sciences Campus, San Juan, PR USA; 3 Department of Microbiology and Immunology, School of Medicine, University of North Carolina, Chapel Hill, NC USA; 4 Department of Molecular Microbiology and Immunology, Saint Louis University, St. Louis, MO USA; 5 Caribbean Primate Research Center, School of Medicine, University of Puerto Rico-Medical Sciences Campus, San Juan, PR USA; 6 Department of Internal Medicine, University of Puerto Rico-Medical Sciences Campus, San Juan, PR USA; 7 Department of Microbiology and Medical Zoology, University of Puerto Rico-Medical Sciences Campus, San Juan, PR USA

**Keywords:** Dengue virus, Live attenuated vaccines

## Abstract

The four dengue virus serotypes co-circulate globally and cause significant human disease. Dengue vaccine development is challenging because some virus-specific antibodies are protective, while others are implicated in enhanced viral replication and more severe disease. Current dengue tetravalent vaccines contain four live attenuated serotypes formulated to theoretically induce balanced protective immunity. Among the number of vaccine candidates in clinical trials, only Dengvaxia is licensed for use in DENV seropositive individuals. To simplify live-virus vaccine design, we identify co-evolutionary constraints inherent in flavivirus virion assembly and design chimeric viruses to replace domain II (EDII) of the DENV2 envelope (E) glycoprotein with EDII from DENV4. The chimeric DENV2/4EDII virus replicates efficiently in vitro and in vivo. In male macaques, a single inoculation of DENV2/4EDII induces type-specific neutralizing antibodies to both DENV2 and DENV4, thereby providing a strategy to simplify DENV vaccine design by utilizing a single bivalent E glycoprotein immunogen for two DENV serotypes.

## Abstract (web summary)

Here, the authors report a live chimeric DENV2/4 EDII virus, encoding DENV2 and DENV4 neutralizing epitopes, that replicates efficiently in primates and simultaneously elicits neutralizing DENV2 and DENV4 type-specific antibodies, providing domain-specific diagnostic reagents and simplified vaccine strategies.

## Introduction

The four serotypes of Dengue virus (DENV1-4) co-circulate in tropical regions, wherever humans co-exist with *Aedes aegypti* or *Aedes albopictus* mosquito vectors^1,2^. DENV1-4 are positive-sense RNA viruses belonging to the Flavivirus genus and cause an estimated ~300-400 million infections each year^3^. While many DENV infections are asymptomatic, symptomatic infections can range from mild dengue fever to severe dengue shock syndrome and death^4^. Infection with a single DENV serotype rarely confers long-lasting immunity to the remaining three serotypes. Rather, a primary infection will generally confer life-long, type-specific (TS) immunity to the infecting serotype, while immunity to the remaining three serotypes is typically short-lived^5^. Importantly, individuals experiencing a secondary infection with a new serotype are at greater risk of developing severe dengue hemorrhagic fever or shock syndrome compared to individuals experiencing primary infections. Although mechanistically complex, in some people DENV serotype cross-reactive (CR) and non-neutralizing antibodies induced by primary infections have been associated with enhanced viral replication and more severe disease during secondary infection^6–9^. This phenomenon has been named antibody-dependent enhancement (ADE), a leading concern in the development of dengue vaccines^8,10,11^.

The icosahedral envelope of DENV consists of 180 tightly packed E glycoproteins, which are critical for viral attachment and entry into cells and the main target of human neutralizing and protective antibodies in individuals who have recovered from primary and secondary DENV infections. The epitopes of several human TS and CR neutralizing antibodies (NAbs) have been mapped to quaternary structure epitopes with footprints that span two or more adjacent E glycoproteins on the viral envelope^12–14^. Recent studies have demonstrated the complexity and relative locations of neutralizing epitopes that reside in DENV3 E domains I, II, and III (EDI, EDII and EDIII)^15^. In the icosahedral envelope, EDI is located at the three-fold axis, EDII is located at the 2 fold axis and EDIII constitute the five-fold axis^16^. We and others have defined which NAb epitopes and antigenic sites are targeted by polyclonal serum-neutralizing antibodies in individuals who have recovered from DENV infections or received candidate vaccines^13,15,17–23^.

The most advanced DENV vaccines are tetravalent live virus vaccines that are formulated with representative strains from each serotype^24,25^. Vaccination goals are designed to induce robust durable immunity to all four serotypes of DENV simultaneously, thereby minimizing the possibility for severe disease enhancement following exposure events. These vaccines are very effective in seropositive individuals, however, in naïve human populations, it has proven difficult to achieve balanced protective immune responses against all 4 serotypes, likely due to the unbalanced replication of vaccine serotypes^19^. Unbalanced immunity induced by the Dengvaxia vaccine was associated with the variable efficacy between serotypes and an increased risk of severe dengue disease upon subsequent exposure to WT DENVs^10,26^. TAK-003 is dominated by the replication and immunogenicity of the serotype 2 component^19,21,22^. In the phase three efficacy trial, the vaccine was most efficacious over the long term (>24 months) against serotype 2 in baseline seronegative children^27^. While the NIH/Merck vaccine appears to be better balanced with respect to vaccine immunogenicity, no efficacy results have been reported for this vaccine^18^.

Given the technical challenges associated with achieving equal replication of 4 live virus vaccine components, one potential solution is to incorporate type-specific epitopes from two different DENV serotypes into a single attenuated vaccine virus. This strategy is feasible because recent epitope mapping studies indicate that many neutralizing epitopes on serotypes DENV2 and DENV4 are located at unique non-overlapping sites in the viral envelope and are interchangeable^13,17^. As major neutralizing monoclonal antibody (mAb) epitopes have been mapped to distinct sites on DENV2 (2D22 on EDII/EDIII and 3F9 on EDI)^14,17^ and DENV4 (NAbs 126 and 131 on EDII)^13^, we developed a recombinant DENV whose E protein contains ED segments of DENV2 and DENV4 displaying these type-specific NAb epitopes. Here we report on the properties of the DENV2/4EDII chimeric E protein virus as well as its immunogenicity when used as a live vaccine immunogen in non-human primates (NHPs). The chimeric DENV2/4 EDII recombinant vaccine virus replicated in NHPs and elicited type-specific NAbs to both DENV4 and DENV2. Our results articulate a simpler strategy to one day develop bivalent live dengue vaccines that induce independent immunity to multiple serotypes.

## Results

### Design and recovery of DENV2/4 chimeric virus

**Fig. 1:**
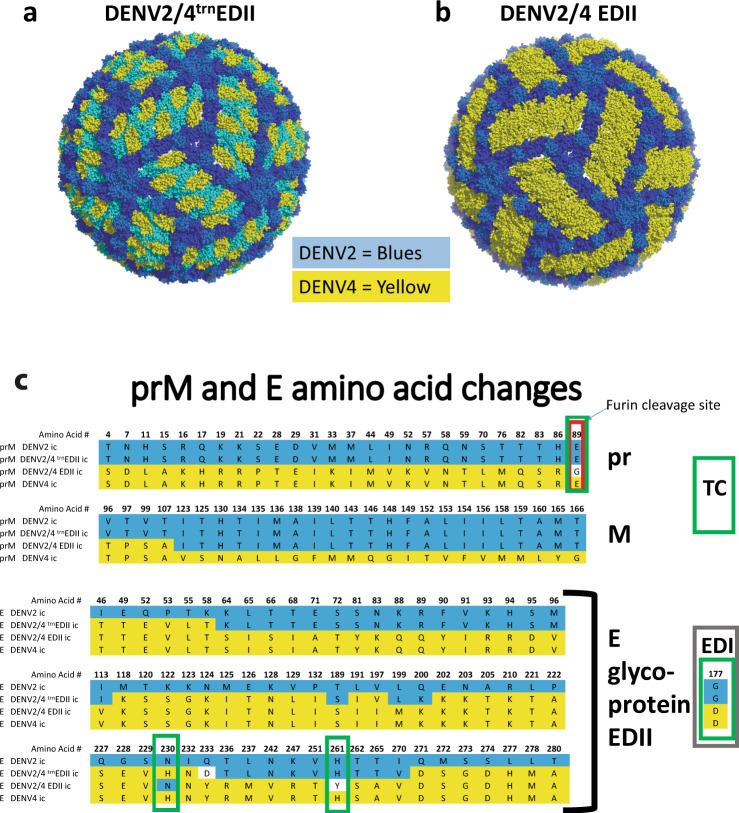
Molecular models of Virions. **a** PyMOL software-generated representations of changed residues. DENV2/4^trn^EDII virion with a truncated EDII transplanted DENV4 region in yellow. DENV2 backbone in shades of blue, EDI - dark blue, EDII – light blue, EDIII – medium blue. **b** DENV2/EDII virion with transplanted DENV4 EDII in yellow. DENV2 backbone in shades of blue, EDI - dark blue, EDIII – medium blue. **c** Amino acid changes to prM and E glycoproteins. Chimeric prM is shown in comparison with parental DENV2 and DENV4 prMs. In the chimeric DENV2/4 prM, amino acids 1-107 are from DENV4 and amino acids 108-166 are from DENV2 with the exception of E89G, which is an essential tissue culture adaptation found in recovered DENV2/4EDII. Chimeric E glycoproteins from DENV2/4^trn^EDII and DENV2/4EDII with their respective tissue culture mutations are shown in comparison with parental DENV2 and DENV4 sequences. DENV2 amino acids are shown in blue and DENV4 are yellow. Amino acids unique to the chimeras have white background. All amino acids not shown are homologous to DENV2 and DENV4. The furin cleavage site in prM is denoted by the red box. Tissue culture (TC) changes are denoted by green boxes. The single amino acid change in EDI is shown in a brown box.

Using human mAbs isolated from individuals who had recovered from primary DENV2 or DENV4 infections, we have identified major DENV2 TS neutralizing antibodies and epitopes that reside in EDI and EDIII, and DENV4 TS NAbs and epitopes that localize to EDII^13,17^. For these studies we created a DENV2/4M14 chimera that displayed DENV4 epitopes on EDII recognized by DENV4 TS NAbs 126 and 131 on a DENV2 backbone^13^. We expanded the DENV4 EDII transplanted region to include the EDI-EDII hinge area in a new chimera, DENV2/4^trn^EDII (Fig. 1a), which was stable and grew to 10^6^ ffu/ml in Vero81 cells. However, attempts to transplant the entire DENV4 EDII domain into DENV2 were unsuccessful.

**Fig. 2:**
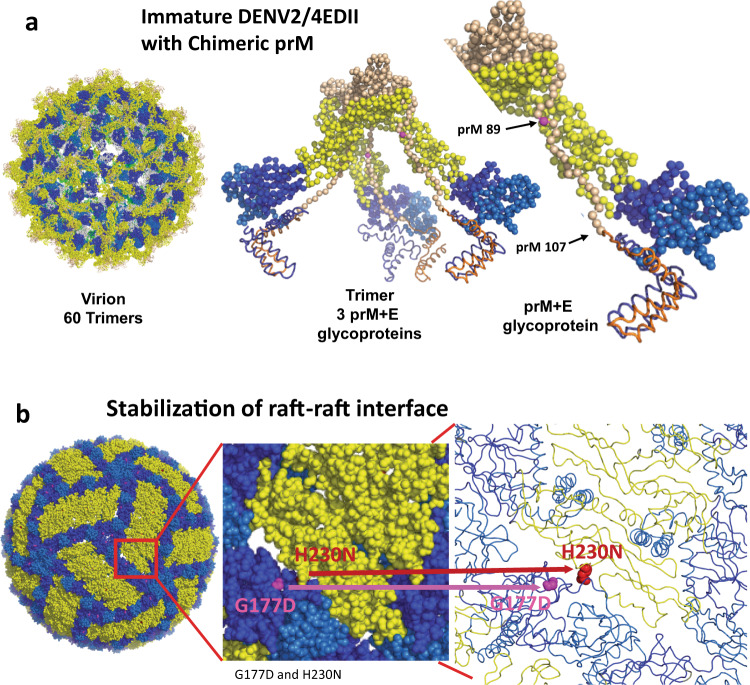
Molecular models of chimeric prM and Tissue Culture Adaptive Mutations. **a** The immature DENV2/4EDII virion, which is composed of 60 prM+E trimers, is shown beside a trimer of prM+E and a monomer of prM+E glycoproteins. The E glycoprotein is represented in dark blue (EDI), yellow(EDII) and medium blue(EDIII) spheres with the stem and membrane alpha-helixes in dark blue ribbon. The DENV4 portion of the chimeric prM, aa 1-107, is shown in tan spheres and the DENV2 portion, aa 108-166, in orange ribbon. The TC change at aa89 is denoted by a pink sphere. **b** The mature DENV2/4EDII virion is composed of 30 rafts, each holding 3 E glycoprotein homodimers. The raft-raft boarder is enlarged in first a spheres and then a ribbon depiction. TC changes G177D (pink) and H230N (red), which converted a DENV2-G to a DENV4-D at 177 and a DENV4-H to a DENV2-N are shown.

**Fig. 3:**
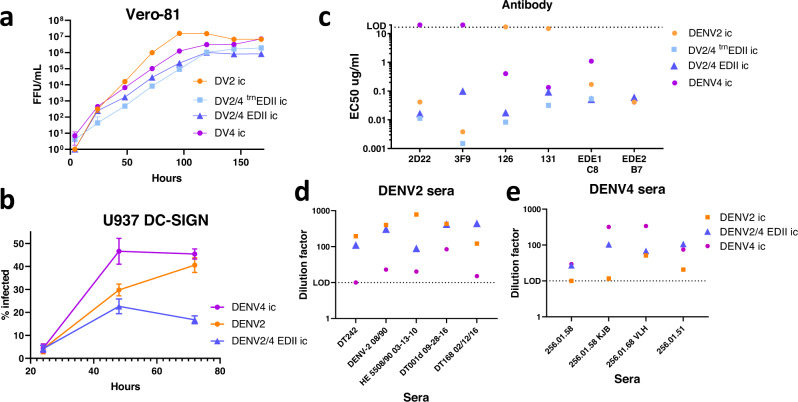
DENV2/4 chimera Replication and Antigenicity. **a** Both DENV2/4 chimeras are slightly attenuated compared to parental DENV2 and DENV4 but replicate efficiently in Vero-81 cells. Mean + /- SD of triplicate values shown. **b** DENV2/4EDII replicates efficiently human macrophage cell line U937 + DC-SIGN but is slightly attenuated compared to parental DENV2 and DENV4. Mean + /- SD of triplicate values shown. **c** Both DENV2/4 chimeras are neutralized by DENV2 mAbs 2D22 and 3F9 as well as DENV4 mAbs 126 and 131. Cross-reactive neutralizing epitopes for hmAbs1 EDE1-C8 and EDE2-B7 are also retained. EC_50_ values from Vero-81 focus reduction neutralization test (FRNT). EC_50_ value denotes the amount of hmAb needed to neutralize 50% of the virus. **d** The DENV2/4EDII chimera contains epitopes targeted by DENV2 primary sera. Vero-81 FRNT EC_50_ values of DENV2ic, DENV4ic and DENV2/4EDII. **e** The DENV2/4EDII chimera contains epitopes targeted by DENV4 primary sera. Vero-81 FRNT EC_50_ values of DENV2ic, DENV4ic and DENV2/4EDII.

As complex interactions between prM and E glycoprotein are important determinants of Flavivirus maturation^28^, preserved DENV2 and DENV4 chimeric prM-E protein-protein interaction networks might provide essential functions in maintaining DENV2/4EDII viability. Using structure-guided modeling, we tested this hypothesis by engineering a chimeric DENV2/4 prM that would maintain interactions with the DENV4 residues on EDII, and DENV2 E residues on EDIII, stem and transmembrane regions of E protein, respectively^28^. In the chimeric prM, the N-terminal aa 1-107 of DENV4 prM precede fifteen residues conserved in DENV2 and DENV4 and are coupled with the remainder of the DENV2 prM protein, aa 123-166 (Fig. 1c). This design is predicted to allow DENV4 EDII and pr protein residues to interact in immature particles while preserving the DENV2 E and M protein interactions in mature particles (Fig. 2a), as well as encoding DENV2 residues at the 3-and 5-fold axis, and DENV4 residues at the 2-fold axis of the virion (Fig. 1b). Using experimental evolution by serial passage in cell culture, the DENV2/4EDII chimeric virus also evolved four tissue culture mutations, one in prM (E89G) and three in the E protein (G177D, H230N, H261Y). The prM (E89G) mutation was present in newly recovered virus and was localized in the prM furin cleavage site. Multiple attempts to rederive this chimeric virus without this mutation were unsuccessful so the mutation appears essential for recovery of the DENV2/4EDII chimeric virus, although the mutation does not change the Pi-Tou score for furin cleavage,13.2573^29,30^. DENV2/4EDII is more mature than DENV2 and similar to DENV4, as inferred by prM to E glycoprotein ratios of 0.40, 2.83 and 0.29 respectively (Supplementary Fig. 1A). In contrast, other mutations, that appeared by passage 5, were predicted to stabilize inter-raft interactions between the DENV2 EDI and DENV4 EDII domains (Fig. 2b). Interestingly, G177D converts a DENV2 residue into a DENV4 residue while H230N converts a DENV4 residue into a DENV2 residue in the chimeric E glycoprotein. The H230N mutation is proximal to the lateral ridge from the neighboring raft while G177D is near EDII of the neighboring raft. The H261Y mutation resides at the EDII:EDII interface of the E dimer in a short alpha helix, which intimately interacts with the N terminus of the chimeric M protein which now contains DENV4 residues. Modification of these histidine residues might play a role in pH sensing required for membrane fusion^31^. The Vero 81 adapted DENV2/4EDII grows to titers of over 10^6^ ffu/ml in Vero 81 cells (Fig. 3a). Additionally, DENV2/4EDII infects and replicates efficiently in a human continuous macrophage cell line, U937 + DC-SIGN (Fig. 3b). The ability of this DENV2/4EDII chimera to replicate efficiently in both Vero 81 and U937-DC-SIGN cells supports its potential as a vaccine candidate to test the bivalent vaccine theory. A purified passage 7 stock, validated by sequence to have high penetrance (>94%) of the adaptive mutations and low secondary background mutations, was used in subsequent studies in vitro and in vivo (Supplementary Fig. 1B).

### DENV 2/4 recombinant virus had DENV2 and DENV4 antigenic properties

The DENV2/4EDII chimera is designed to be neutralized by type-specific antibodies targeting both DENV4 and DENV2. As predicted, the DENV2/4EDII and DENV2/4 ^trn^EDII (truncated EDII) chimeras were neutralized by two DENV2 specific hmAbs; the 2D22 antibody binds a quaternary EDII/EDIII epitope while the 3F9 antibody binding epitope resides mostly in EDI (Fig. 3c)^14,32^. Depending on the donor sera, the mAbs 2D22 and 3F9 epitope domains are targeted by up to 80% and 54% of the polyclonal neutralizing antibody response after natural DENV infection, respectively, with some sera targeting both^17^. The partial loss of neutralization by mAb 3F9 against the DENV2/4 EDII chimera, as compared to the DENV2/4 ^trn^EDII and DENV2, likely reflects tissue culture mutation G177D in EDI. This residue not only lies within the 3F9 epitope footprint^17^, but earlier chimeras that retained G177 had similar 3F9 neutralization EC50 titers as DENV2. Importantly, the panDengue cross-reactive EDEI C8 and EDE2 B7 neutralizing epitopes, found on all DENV serotypes, retained full neutralization potency against both the DENV2/4 ^trn^EDII and DENV2/4EDII viruses (Fig. 3c).

In both DENV2/4 chimeras, the DENV4 EDII epitopes appeared structurally intact as indicated by efficient neutralization by DENV4 hmAb 131 and a slightly increased neutralization phenotype by hmAb 126, which binds an epitope near the EDI/II hinge region adjacent to the DENV4 transplanted residues^13^. Although speculative, the DENV2/4 chimera may have subtle structural changes in topology, which enhanced the presentation for the DENV4 hmAb 126 epitope.

We next used convalescent human primary DENV2 and DENV4 sera to evaluate neutralization of the DENV2/4 EDII chimera and the two parental serotypes (Fig. 3d). Three of five DENV2 primary sera neutralized the DENV2/4EDII recombinant virus at similar concentrations as the parental DENV2 serotype, fully preserving the polyclonal neutralization phenotype. One sera neutralized the parental DENV2 virus 9-fold better than the chimera potentially revealing the presence of undiscovered DENV2 neutralizing epitopes lost in the DENV2/4EDII virions. In contrast, one patient sera neutralized the chimera 4-5 fold better than the parental DENV2, which may reflect more efficient presentation of cryptic and/or key-neutralizing epitopes in the virion. Using this collection of primary sera, our data confirms earlier studies where most responses targeted EDI and/or EDIII epitope domains^17,18^. Two of four DENV4 primary sera neutralized DENV2/4EDII as well as the parental DENV4 and two neutralized the parental DENV4 better than the chimera, indicating that some DENV4 epitopes were not presented in the chimera.

### DENV2/4EDII replication is attenuated in primates

The DENV2/4EDII chimera envelope surface area is approximately equally distributed between DENV2 and DENV4, replicates well in both Vero-81 and U927 cells and presents DENV2 and DENV4 epitopes that are recognized by hmAbs and polyclonal immune serum. Consequently, we next determined whether vaccination with the DENV2/4EDII chimera could induce type specific immune responses in primates that target both DENV2 and DENV4.

**Fig. 4:**
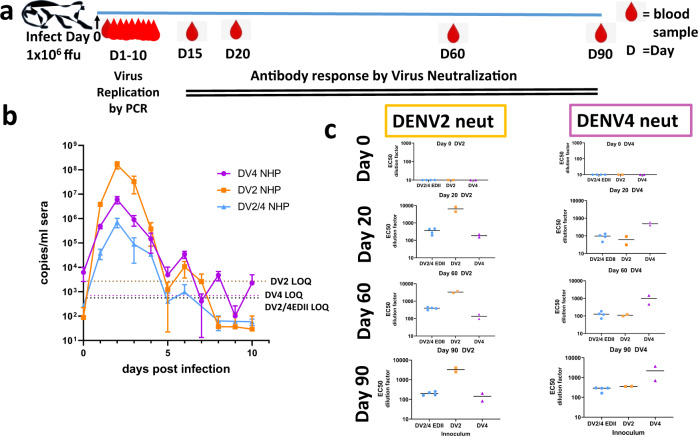
DENV2/4EDII chimera replicates efficiently in primates and induces neutralizing antibodies. **a** Timeline for inoculation of NHP with 10^6^ ffu DENV2ic(2NHP), DENV4ic(2NHP) or DENV2/4EDIIic(4NHP) and blood collection for quantitative reverse transcript-polymerase chain reaction (qrt-pcr) and FRNT analysis. **b** Replication kinetics of DENV2/4EDII is temporally similar to parental DENV2 and DENV4. Average copies/ ml sera, as determined by qrt-pcr, is shown for DENV2, DENV4 and the four DENV2/4EDII inoculated NHP. Copies/ml was measured in triplicate for each NHP, then combined with other NHP within the treatment group This data graphed by individual NHP can be seen in supplementary Fig. 2A. Hashed line represent the limit of quantitation (LOQ) for each virus. Mean values + /- SD are shown. **c** DENV2/4EDII sera neutralized DENV2 and DENV4. Individual Vero-81 FRNT EC_50_ values are shown for each NHP with a bar representing the median value. EC_50_ values were calculated using 8 point curves performed in duplicate.

Rhesus macaques, nonhuman primates (NHP), with no prior exposure to DENV were inoculated subcutaneously with 1 × 10^6^ ffu of DENV2/4EDII (*n* = 4) or DENV2 (*n* = 2) or DENV4 (*n* = 2), from deep sequenced verified stocks (Fig. 4a). Body temperature was monitored and blood was collected on days 0, 1–10, 15, 20, 30, 60 and 90 postinoculation. Virus titer in the blood was accessed by quantitative RT-PCR. The replication of the DENV2/4EDII chimera was attenuated as compared with DENV2 and DENV4, although all 3 displayed similar temporal growth kinetics that peaked on day 2 and cleared by day 10 postvaccination. Depending on the animal, DENV2 titers peaked at 1-2 × 10^8^ genome copies /ml sera, DENV4 peaked at 4-8 × 10^6^ genome copies /ml sera and DENV2/4 EDII chimera peaked at 0.2-1 × 10^6^ genome copies /ml sera (Figs. 4b and S2). Each virus elicited a humoral immune response that could be tracked by Vero-81 foci reduction neutralization (FRNT) assay (Fig. 4c). All 8 NHP were naïve to DENV on day 0 and by day 15 postinoculation, all 8 NHP showed high levels of neutralizing anti-DENV antibodies that were stable thru day 90. As expected, DENV2 inoculated primates had the highest DENV2 neutralizing titers. Those inoculated with DENV4 had the highest DENV4 neutralizing titers. Importantly, NHP inoculated with DENV2/4EDII had DENV2 and DENV4 neutralizing titers above 200 after one dose. While this is above or comparable to titers seen in NHP with 2 doses of other vaccine formulations^33,34^, overall levels of neutralizing antibodies to a particular serotype are not always a reliable measure of protection.

### DENV2/4 EDII elicits type-specific response to DENV2 and DENV4

**Fig. 5:**
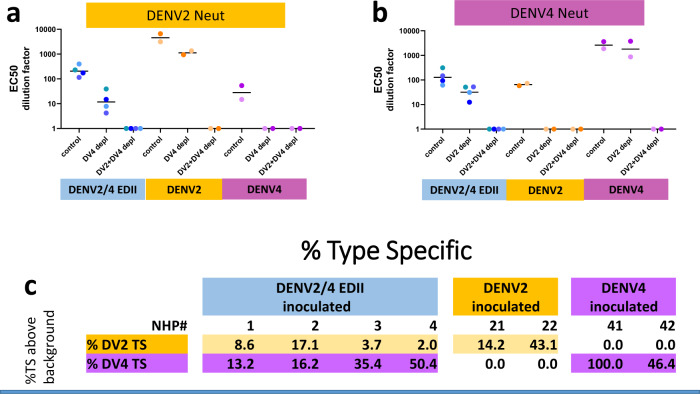
DENV2/4EDII induced Type Specific (TS) antibodies to both parental DENV2 and DENV4 strains. **a** Type-Specific Abs to DENV2 were identified in DENV2 sera and DENV2/4EDII sera but not DENV4 sera. Sera was depleted by BSA(control), DENV2 or DENV2 + DENV4 virions. Background (DV2 + DV4 depl) subtracted Vero-81 FRNT EC_50_ values are shown. Individual EC_50_ values are shown with a bar at the median value. Neutralization curves for each EC_50_ are shown in supplementary Fig. 4. **b** Type-Specific Abs to DENV4 were identified in DENV4 and DENV2/4EDII sera but not DENV2 sera. Sera was depleted with BSA(control), DENV4 or DENV2 + DENV4 virions. Background (DV2 + DV4 depl) subtracted Vero-81 FRNT EC_50_ values are shown. Individual EC_50_ values are shown with a bar at the median value. Neutralization curves for each EC_50_ are shown in supplementary Fig. 5. **c** Table shows the percent of Type-Specific Abs to DENV2 or DENV4 as compared to the BSA-depleted controls. 100*(heterologous depleted sera EC50 – DENV2 + DENV4 depleted sera EC50)/(BSA depleted sera EC50 – DENV2 + DENV4 depleted sera EC50).

Type-specific neutralizing antibodies (targeting unique epitopes on a serotype) are not sensitive to virus maturation state and are thought more predictive of in vivo protection than total levels of neutralizing antibody^35,36^. Therefore, we measured type-specific antibody responses. The 90-day sera were depleted of cross-reactive antibodies using beads coated in BSA, DENV2, DENV4 or DENV2 + DENV4 and then tested by ELISA and FRNT assays (Fig. 5 and Supplementary Fig. 3). DENV2 TS responses were defined as those antibodies that remained in the DENV4-depleted sera above background (Fig. 5a and Supplementary Fig. 4). The two DENV2 inoculated NHP had the highest level of DENV2 TS antibody titers, which represented 14% and 43% of the control depleted titers (Fig. 5c). All four DENV2/4EDII-inoculated NHPs also had DENV2 TS antibodies, albeit in lower titers, with TS responses representing 9%, 17%, 4% and 2% of control depleted titers of 174, 229, 115 and 399 DF, respectively (Figs. 5a and c, Supplementary Table 1). As expected, the DENV4 sera contained CR antibodies but no TS DENV2 antibodies. DENV4 TS antibodies were defined as those left in the DENV2-depleted sera above background. The two DENV4 inoculated NHP developed high titers of DENV4 TS antibodies, representing 100% and 46%, respectively of the control serum titers (Fig. 5b, c and Supplementary Fig. 5). Sera from all four DENV2/4EDII chimera inoculated NHP contained TS DENV4 antibodies that represented 13, 16, 35 and 50% of control sera, respectively (Fig. 5b and c). Only the four NHP that were inoculated with DENV2/4EDII developed TS antibodies against both DENV2 and DENV4, demonstrating a clear bivalent response (Fig. 5c).

**Fig. 6:**
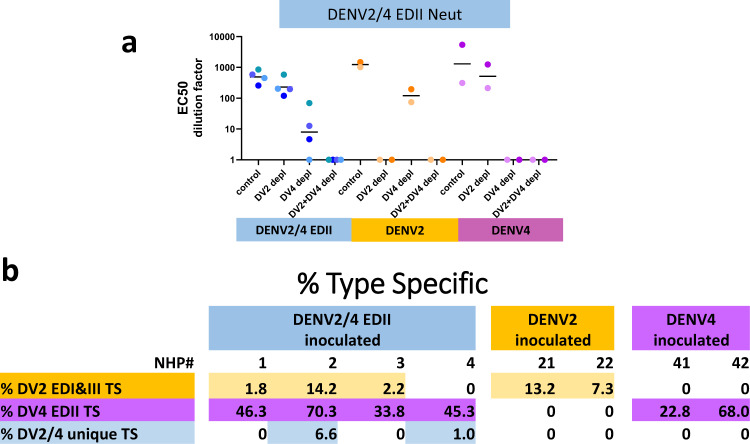
Identification of DENV2/4EDII TS Abs. **a** Type-Specific Abs to DENV2/4EDII were identified in DENV2, DENV4 and DENV2/4EDII sera. Sera was depleted with BSA(control), DENV2, DENV4 or DENV2 + DENV4 virions as described in the Methods. Background (DV2 + DV4 depl) subtracted Vero-81 FRNT EC_50_ values are shown. Individual EC_50_ values are shown with a bar at the median value. Neutralization curves for each EC_50_ are shown in supplementary Fig. 6. **b** The table shows the percent of Type-Specific Abs to DENV2 EDI + III or DENV4 EDII as compared to the BSA depleted controls. 100*(heterologous depleted sera EC50 – DENV2 + DENV4 depleted sera EC50)/(BSA depleted sera EC50 – DENV2 + DENV4 depleted sera EC50).

We also tested the depleted sera against the DENV2/4EDII chimera to show that sera from NHP infected with DENV2 and DENV4 targeted the epitopes presented in the chimeric DENV2/4 EDII (Fig. 6a and Supplementary Fig. 6). DENV2 sera contained high titer TS NAbs targeting EDI and EDIII of the chimera with EC50s significantly above background. Similarly, both of the DENV4 NHP had high titer TS NAbs targeting EDII of the chimera. Indeed the TS NAbs targeting these domains of the chimera were higher in the DENV2 and DENV4 sera than in the chimera sera. This is not surprising since the parental strains grew to higher titers than the chimera in the NHP. As expected, the chimera sera had higher EC50s against itself than the parental strains with TS NAbs against both DENV2 EDI&III and DENV4 EDII.

Because unique epitopes might be presented during DENV2/4EDII infection as compared to wildtype parental strains, TS responses in DENV2 + DENV4 depleted sera from DENV2/EDII infections were conducted and significant levels of DENV2/4EDII specific polyclonal responses were detected in one of the four vaccinated animals. NHP2 retained an EC50 of 81 against the chimera after depletion with DENV2 + DENV4 (Supplementary Table 1), indicating that 7% of the DENV2/4EDII neutralizing titer is vaccine strain specific, while 14% is directed against DENV2 and 70% against DENV4 (Fig. 6b). NHP4 had negligible levels of unique DENV2/4EDII NAbs while NHP1 and 3 had none, seeming to indicate TS antibodies were mainly targeted to the homologous domains of each serotype and not to the border areas between the serotypes.

### In vitro antibody-dependent enhancement of DENV by wildtype and chimeric NHP sera was similar

Because enhancement of DENV by non-neutralizing CR antibodies is a significant problem, we compared the activity of wildtype and chimera sera to enhance infection of K562 cells in vitro. DENV2 sera and DENV2/4EDII sera both enhanced DENV4 similarly (Supplementary Fig. 7A). Additionally, DENV4 sera and DENV2/4EDII sera both enhanced DENV2 similarly (Supplementary Fig. 7B). As all sera enhanced DENV2 or DENV4 infection with similar kinetics and at similar concentrations, vaccination with chimeric surface glycoproteins did not enrich this phenotype over wildtype control serum.

## Discussion

Effective flavivirus vaccines exist for Japanese Encephalitis, Tick Borne Encephalitis virus and Yellow Fever virus, yet the only licensed DENV vaccine, Dengvaxia, is not recommended for use in naïve populations due to an increased risk of severe DENV after infection^10^. DENV vaccine development is further hampered by uncertain correlates of protective immunity, which are confounded by the presence of breakthrough infections in individuals with relatively high titers of neutralizing antibodies and T-cell responses^17,18,37–39^. The basis for breakthrough infections is complex, perhaps mediated by T cell responses, genotype variation within serotypes, limited protection by cross-reactive antibodies, and an incomplete understanding of the role of ED domain and type-specific neutralizing responses across serotypes. In addition to Dengvaxia, two additional live tetravalent attenuated vaccines, DENVAX(Takeda) and TV003(NIH), are in phase 3 trials^24,40^. Recently, DENVAX was approved for use in Indonesia and by the European Medicines Agency for use in Europe^41^ and TV003 was reported by Instituto Butantan to have a preliminary efficacy nearing 80% against DENV1 and DENV2^42^. Early indications point to TV003 achieving improved and balanced immune responses across the 4 serotypes, however, phase 3 efficacy data for this vaccine is not yet available^18^. Using DENV virion structure-guided reverse genetics, we developed virologic reagents to measure ED-specific neutralizing antibody responses and a novel vaccine platform to promote more balanced bivalent type-specific immune responses after vaccination. By matching serotype-specific prM and E glycoprotein interaction motifs in immature and mature virions, viable chimeras were obtained with bivalent E glycoproteins derived from 2 different serotypes, DENV2 and DENV4. This approach also has the advantage of balancing two serotype responses within one virus and tying the immune response of the two serotypes together. Importantly and after a single vaccine administration, the chimeric DENV2/4EDII recombinant virus elicited TS-neutralizing antibodies to both DENV2 and DENV4 in all NHP. We anticipate that increased dosage and boosters would further enhance these type-specific responses.

Structural studies have identified interacting residues that stabilize E glycoprotein dimer-dimer interaction and the formation of complex E glycoprotein rafts^43,44^. The design of DENV2/4EDII, encoding EDII from a different serotype, revealed additional structural constraints in the assembly of DENV E glycoprotein monomers into dimers and rafts, including a role for matching prM-E glycoprotein interaction domains in the production of infectious virions. In fact, recombinant virus viability was dependent upon matching chimeric DENV4 prM and DENV4 EDII interaction domains, while preserving DENV2 prM EDIII/transmembrane interactions^28^. These data support earlier studies with DENV virus-like particles (VLP), where prM and E interactions were essential for efficient VLP production, while also playing important roles in the maturation and suppression of fusion function during egress^16,45^. In chimeric E recombinant virus, experimental evolution selected for mutations that appear to reestablish efficient interactions between EDII and EDI in adjacent monomers from neighboring dimers or rafts. Modeling predicts that the G177D and H230N mutations restore appropriate serotype-specific interactions and stability along this raft-to-raft boarder where DENV2 EDI-EDIII and DENV4 EDII interact, enhancing virus viability and growth. The H261Y mutation in E resides on the underside of the E glycoprotein and is unlikely to affect antigenicity although it may affect dimer/dimer stability.

Previously, we successfully transplanted EDI (3-fold axis) and EDIII (5-fold axis), but not EDII sequences from DENV3 into a DENV1 E glycoprotein backbone^15,21^. In contrast, the DENV2/4EDII recombinant virus encodes an E glycoprotein whose 3-and 5-fold axis were derived from DENV2, coupled with a DENV4 2-fold axis, implanting critical DENV4 EDII neutralizing epitopes into DENV2. While demonstrating the portability of all three E glycoprotein ED residues between serotypes, we have also used live bivalent viruses to measure epitope and domain-specific responses to various DENV serotypes. Using the DENV2/4 EDII chimera, we confirmed that the largest fraction of the DENV4 human polyclonal responses after primary infection targets the DENV4EDII region. Our data support the hypothesis that the DENV4EDII is prominently targeted after primary DENV4 infection while epitopes in EDI and spanning EDII/EDIII are primary targeted after DENV2 infection. In the future, simultaneous measurement of EDI, EDII and EDIII TS antibody responses to a serotype would provide a more comprehensive picture of individual variation and domain-specific neutralizing antibody response patterns after primary and secondary infections, and whether domain cross-reactive or type-specific responses correlate with protection and/or breakthrough infections.

Chimerized, viral surface glycoproteins represent a novel strategy to produce broad, cross-neutralizing live virus and recombinant virus vectored vaccines against influenza virus and Sarbecoviruses^46,47^. Importantly, this approach has been shown to elicit type-specific neutralizing antibody and broad, cross-neutralizing antibody responses that protect from heterologous challenge in vivo. This is the first chimeric DENV to elicit TS antibodies to two different serotypes following a single infection. Using DENV serotype-specific and cross-reactive monoclonal antibodies, we demonstrate that the major DENV2 and DENV4 TS and cross-reactive neutralizing epitopes are preserved in DENV2/4EDII virions. DENV2/4EDII displayed an attenuated growth phenotype in primates that was comparable with current DENV vaccines under development^33,34^ and comparable neutralizing responses against both DENV2 and DENV4. Since much of this response was associated with cross-reactive antibodies, we used depletion studies to demonstrate type-specific immune responses against both intended targets of the vaccine, DENV2 and DENV4. While infection of primates with either DENV2 and DENV4 wildtype viruses clearly induced higher levels of total and TS neutralizing antibody than the chimeric DENV2/4EDII, our studies provide proof of principal that a bivalent TS neutralizing antibody response can be elicited by a chimerized, live DENV recombinant virus in primates. Total neutralizing titers appeared to correlate well with virus growth efficiency since DENV2 and DENV4 replicated to 1000 and 10-fold higher titers than DENV2/4EDII infection, respectively, potentially explaining the reduced neutralizing titers seen in the vaccine strain. We imagine several strategies for boosting type-specific responses, including increasing the inoculum dose and/or providing a booster inoculation with the same or complementary DENV4/2EDII immunogen. Alternatively, chimeric DENV immunogens could be delivered as mRNA nanoparticle or stabilized recombinant E glycoprotein vaccines plus adjuvant^46^. Importantly, chimeric viruses that induce TS immunity to two different DENV serotypes may circumvent strain-specific immune dominance problems associated with live tetravalent virus vaccine use.

Structure-guided synthetic gene design, reverse genetics and experimental evolution provide numerous opportunities for assembling cis and transregulatory multicomponent protein machines that regulate virion maturation, stability, viability and immunogenicity. We show that the extensive genetic manipulations used to engineer a bivalent DENV E glycoprotein are associated with fitness costs that attenuate virus pathogenesis and limit in vivo replication in primates. Moreover, we anticipate that the DENV2/4EDII chimera would require a matching DENV3/1 EDII or DENV1/3 EDII chimera, designed as a balanced, two-component vaccine that maximizes a tetravalent response in individuals. Balancing a two-component vaccine may be easier than a four-component system, but the outcome of a balanced immune response across the four serotypes remains an elusive goal. Given the unbalanced immunity elicited in some people by leading tetravalent vaccines^19^, we speculate that bivalent vaccine formulations might also be used in conjunction with current vaccines to provide a means of enhancing and balancing response as a prime-boost combination strategy or as a boost to existing vaccines.

The characterization of the DENV2/4 chimera and subsequent immunogenicity studies were undertaken to test if infection with the chimera would lead to the immunodominance of epitopes from a single serotype or if epitopes from both serotypes are able to elicit serotype-specific responses. Our results demonstrate that epitopes from both serotypes independently stimulate antibodies. Limitations in the study include the small number of NHPs used to test immunogenicity. Additional studies are needed to fully characterize immunogenicity and feasibility of chimeric E protein vaccines. While the chimeric virus was designed to create large surface areas that preserve type-specific antibody responses in a domain-specific pattern, chimeric E glycoproteins may also present novel epitopes that suppress responses to native epitopes on one or both serotypes. The levels of type-specific neutralizing antibodies induced by the DENV2/4 chimera were low. Future studies will prioritize improving immunogenicity and the assessment of short and long-term protection against vaccine-matched and mismatched genotypes. Although growth was attenuated in primates, studies are also needed to confirm the attenuated replication, pathogenic and safety phenotypes of the chimeric viruses in humans. Nevertheless, the ability of a chimeric DENV to elicit TS antibodies to both DENV2 and DENV4 warrants continued investment for future use.

## Methods

### Ethics statement

All procedures were reviewed and approved by the Institute’s Animal Care and Use Committee at Medical Sciences Campus, University of Puerto Rico (IACUC-UPR-MSC) and performed in a facility accredited by the Association for Assessment and Accreditation of Laboratory Animal Care (AAALAC) (Animal Welfare Assurance number A3421; protocol number, 7890116). Procedures involving all study animals were approved by the Medical Sciences Campus, UPR IACUC and were conducted in accordance with USDA Animal Welfare Regulations, *the Guide for the Care and use of Laboratory Animals* and institutional policies. In addition, steps were taken to lighten sufferings in accordance with the recommendations of the Guide for the Care and use of Laboratory Animals (8th edition), Animal Welfare Act and the Public Health Service (PHS) Policy on Humane Care and Use of Laboratory Animals. All procedures were conducted under anesthesia by intramuscular injection of ketamine at 10–20 mg/kg^−1^ of body weight, as approved by the IACUC. Anesthesia was delivered in the caudal thigh using a 23-Gauge sterile syringe needle. During the period of the entire study, the macaques were under the environmental-enrichment program of the facility, also approved by the IACUC. In addition, steps were taken to lighten sufferings, including the use of anesthesia and method of sacrifice if appropriate, following the Weatherall report” “The use of non-human primates in research: http://www.acmedsci.ac.uk/more/news/the-use-of-non-human-primates-in-research/.

### Cell lines and viruses

Vero-81 cells (ATCC# CCL-81) were maintained in Dulbecco’s modified Eagle’s/Ham’s F-12 50/50 Mix (DMEM/F-12 50/50) supplemented with non-essential amino acids (NEAA), glutamine and sodium bicarbonate (Vero cell medium) at 37 °C, media was supplemented with 5% fetal bovine serum (FBS) and penicillin/streptomycin antibiotics. U937 cells expressing DC-SIGN (dendritic cell-specific intercellular adhesion molecule-3-grabbing nonintegrin), a known DENV attachment factor, were maintained as suspension cell cultures at 37 °C with 5% CO_2_ in RPMI 1640 (Gibco) supplemented with 1% non-essential amino acids, 1% penicillin and streptomycin, and 5% fetal bovine serum (FBS; HyClone). All viruses used in this study were molecularly rederived by reverse genetics, unless otherwise stated. The rDENV2 clone is based on DENV strain S16803 and the DENV4 molecular clone was based on the sequence of Sri Lankan DENV strain 1992a, and have been previously describe^20,21,48^.

### Generation of recombinant virus

We use a four-component cDNA cloning system in which the DENV genome is divided into four segments that can be replicated separately as plasmids in *Escherichia coli* cells^17,21–23,49^. Purified plasmids are cut with designated restriction enzymes to yield unique type IIS restriction endonuclease cleavage sites that can be ligated simultaneously to yield full-length DENV genome cDNA. A built-in T7 site was used to generate RNA, which was electroporated into C6/36 or Vero-81 cells to recover virus. Virus harvested from the medium is subsequently passaged and sequence verified. To generate additional chimeric rDENV2/4EDII viruses, we systematically increased the numbers and/or locations of amino acid residues from DENV4 EDII transplanted into DENV2. The segment encoding the prM and E proteins was synthesized (BioBasic, Buffalo, NY) and incorporated into full-length assembled DNA genomes and transcribed. Then, the genome-length RNAs were electroporated into Vero-81 cells to generate viable recombinant rDENV2/4EDII virus. Recombinant viruses were subjected to full-length sequencing to demonstrate the presence of appropriate subsets of mutations, as previously described^17,20^. Stocks were validated as an inoculum by deep sequencing methods MiniON Real-Time and Illumina Total RNA sequencing, which provided coverage of greater than 30 K and 20 K, respectively. Both methods found that stocks contained no trace contaminants. The DENV2/4EDII chimera was not independently rederived. DENV2/4^trn^EDII is inventoried as DENV2/4 M14 + .

### Vero cell titration and focus assays

For viral titrations, viral stocks were diluted 10-fold serially in Vero cell medium supplemented with 2% heat-inactivated fetal bovine serum (HI-FBS; Hyclone Defined) and 1x antibiotic. The inoculum was added to Vero-81 cells that were seeded into a 96-well plate (2 × 10^4^ cells/well) the previous day and incubated at 37 °C for 1 h, then overlaid with overlay medium (Opti-MEM I Grand Island, NY, with 1% methylcellulose and 2% heat-inactivated FBS). Viral foci were detected at 44 to 48 h after infection, following fixation/permeabilization with 10% buffered formalin/0.01% saponin using primary murine mAbs 2H2 and 4G2 and secondary horseradish peroxidase (HRP)-conjugated goat anti-mouse IgG (Sigma), followed by TrueBlue substrate (KPL). The number and size of foci were analyzed with a CTL Immunospot instrument.

### Vero cell neutralization assays

Neutralization on Vero-81 cells has been described previously^21^. Briefly, monolayers of Vero-81 cells in 96-well plates were inoculated with a virus and antibody or serum mix that had been incubated for 1 h at 37 °C to allow for Ab:virion binding. Following a 1 hr incubation on cells at 37 °C for infection, cells and inoculum were overlaid with overlay medium (see above). Viral foci were detected at 44 to 48 h after infection, following fixation/permeabilization with 10% buffered formalin/0.01% saponin using primary mAbs 2H2 and 4G2^50^ and secondary horseradish peroxidase (HRP)-conjugated goat anti-mouse IgG (Sigma), followed by TrueBlue substrate (KPL). Numbers of foci were analyzed with an Immunospot Analyzer instrument (Cellular Technology Limited). All hmAb neutralization assays were performed as eight-point dilution curves done in duplicate with at least 2 independent experiments. Variable slope sigmoidal dose-response curves are calculated with top or bottom restraints of 100 or 0, respectively. EC_50_ is the concentration of antibody that neutralizes 50% of the virus being tested.

### U937-DC-SIGN growth curve

U937 human monocytic cell line stably transfected with DC-SIGN^51^. A dilution of virus that infects between 8-15% of the U937 cells (previously determined by virus titration) incubated for 2 h at 37 °C. Following incubation, the cells were centrifuged at 252 x *g* for 5 minutes and resuspended in 200 μL RPMI medium. Next, cells were fixed in 4% paraformaldehyde, incubated for 10 min at room temperature, and centrifuged at 252 x *g* for 5 min. Following this, cells were blocked in permeabilization buffer (0.1% saponin, 5% bovine serum albumin in 1X phosphate-buffered saline [PBS]) for 30 minutes at room temperature. Then, cells were incubated with anti-E mAb 4G2 conjugated to Alexa 488, diluted in blocking buffer (0.5% bovine serum albumin and 0.02% sodium azide in 1X PBS) for 25 minutes at room temperature. Finally, cells were washed and resuspended in PBS. Acquisition of the infected cells was performed with a Guava flow cytometer (EMD Milipore) by gating Alexa 488-positive cells (Supplementary Fig. 8). The data were analyzed using analysis with Prism 7.2 software (GraphPad).

### Nonhuman primate studies

Young rhesus macaques (2.5–4 years of age) seronegative for DENV and ZIKV were housed in the CPRC facilities, University of Puerto Rico, San Juan, Puerto Rico. All macaques were male. Animals were infected subcutaneously in the deltoid area with 500uL of virus diluted in PBS, using a dose of 1 × 10^6^ pfu. Pair of animals were infected with DENV2ic or DENV4ic and four animals were exposed to DENV2/4EDII.

Macaques were continuously monitored by trained veterinarians at the Animal Research Center and evaluated twice daily for evidence of disease or injury. Feeding and drinking continued normally during this period. Weights were taken on Day 0 and every other day during the acute infection period (days 1-10). Rectal temperature was taken daily during the acute infection period (days 1–10 and on day 30 p.i.). After the challenge neither symptomatic manifestations nor significant differences in rectal temperature were observed in most of the animals. Only animal NHP2 reached a temperature discreetly above of the normal internal temperature of rhesus macaques (approximately from 38.5 to 39.3)^52^ with peaks of 39.5 °C and 39.7 °C on days 4 and 5 respectively (Supplementary Fig. 2). However the pattern was similar to the temperature behavior reported by our group in rhesus macaque after being exposed to flavivirus^53–55^. Also weight was stable during the challenge period. Blood was collected on days 0, 1–10, 15, 20, 30, 60 and 90 postinoculation. Stocks used for macaques’ immunization were validated as an inoculum by deep sequencing methods using MiniON Real-Time and Illumina Total RNA sequencing. Sera collected was stored at −80 °C and virus titer in the blood was accessed by quantitative rt-pcr (Supplementary Fig. 2).

### Quantitative reverse transcription-polymerase chain reaction

Viral RNA was isolated from 140ul of macaque sera using QIAmp Viral RNA Mini Kit (Qiagen Cat. No. 52904). Sera was lysed in AVL buffer with 40ug/ml of glycogen (Thermo Fisher), RNA was precipitated by addition of ethanol and added to the column. The column was washed in AW1 buffer followed by AW2 buffer. RNA was eluted in 45ul of water and stored at −80 °C. RNA (22ul) was transcribed to cDNA by Superscript III Reverse Transcriptase (Invitrogen P/N 56575) with random primers. Copies of DENV2, DENV4 or DENV2/4EDII were quantitated by qPCR. A single primer/probe set for DENV2 capsid was used to measure DENV2 and DENV2/4EDII, FWD 5’-GGC GTT CCT TCG TTT CCT AA-3’, REV 5’-TCA GCA TCC TTC CAA TCT CTT T-3’, Probe 5’-/56-FAM/TCC CAC CAA/ZEN/CAG CAG GGA TAT TGA/3IABkFQ/−3’ (Integrated DNA Technologies). A separate primer/probe set was used for DENV4 capsid, FWD 5’- TTT GCG GGT CCT TTC CAT −3’, REV 5’- CGG CCT ATC TCC TTC CTA AAT C −3’, Probe 5’-/56-FAM/CCC ACC AAC/ZEN/AGC AGG GAT TCT GA/3IABkFQ/−3’. Equal volumes, 4ul, of each sample was run in triplicate using iTaq Univeral Probes Supermix (Bio-Rad Cat. No. 1725131) in a Roche LightCycler 480. Plasmids, containing the first 2500nt of DENV2 or DENV4 and of known concentration, were used for standard curves for purposes of quantitation.

### Depletion of heterotypic antibodies

Heterotypic antibodies were removed from the macaque sera by incubation with purified whole virion antigen covalently linked to magnetic beads, as reported previously^19^. The properties of Abs in undepleted and Ab-depleted serum are then assessed by ELISA and mFRNT assays. Briefly, Magnetic Dynabeads M280 Tosyl activated (DB) (Invitrogen 14203, 14204) were first covalently linked to the dengue cross-reactive monoclonal antibody 1M7. DB-1M7 complex was blocked with BSA in PBS and incubated for 1 h at 37 °C in separate with purified DENV2, DENV4 or Bovine Serum Albumin (BSA) antigen control at a ratio of 100 ug of antigen per 5 mg DB. After 3 PBS washes the bound antigen was cross-linked to the DB-1M7 complex with 2% PFA for 20 min at room temperature. Serum samples were diluted 1:10 and incubated with the Antigen-DB complex for 1 h at 37 °C with end-over-end mixing under 3 conditions: a) BSA-DB as undepleted control (total Nabs, TS + CR) (UND), b) DENV2 depleted, c) DENV4 depleted, and d) DENV2 + DENV4 (in 1:1 ratio) depleted. Abs bound to beads were magnetically separated from the depleted serum. Three rounds of depletion were usually needed for successful removal of >80% of homologous dengue Abs. DENV2 TS responses were defined as those antibodies that remained in the DENV4-depleted sera above background. DENV4 TS antibodies were defined as those left in the DENV2-depleted sera above background.

### Analysis of TS NAbs

Neut50 titers in the undepleted, homotypic-depleted and heterotypic-depleted sera were determined by mFRNT. The fraction of Abs in the sera that were TS was calculated using the formula: %TS NAbs = [Neut50 HET depletion – Neut50 HOM depletion]/[Neut50 BSA depletion – Neut50 HOM depletion] x 100, as reported earlier^56^.

### ELISA

Antigen capture ELISA was used to confirm efficient depletion of targeted antibodies. Briefly,4G2:2H2 were used to coat the ELISA plate at 4 °C overnight (100 ng/well each). Plates were then blocked with 5% (w/v) NFDM (Labscientific, Thermo Fisher, USA)-Tris-buffered saline (TBS) −0.05% (vol/vol) Tween 20 (blocking buffer) for 1 hr at 37 °C. The antigen was then captured at 100 ng antigen/well for 1 hr at 37 °C. Plates were blocked with 3% (vol/vol) normal goat serum (Gibco, Thermo Fisher, USA)-Tris-buffered saline (TBS) −0.05% (vol/vol) Tween 20 (blocking buffer). Depleted sera were diluted and added to the antigen-coated ELISA plates. Alkaline phosphatase-conjugated secondary Abs were used to detect the binding of sera with p-nitrophenyl phosphate substrate, and reaction color changes were quantified by spectrophotometry at 405 nm.

### Western blot

Supernatant from of DENV 2 and 4 viral stock were diluted with 4x Laemmli Sample Buffer (Bio-Rad) and boiled at 95 ^o^C for 5 minutes. Following SDS-PAGE electrophoresis, proteins were transferred to PVDF membrane and blocked in blocking buffer (5% non-fat milk in PBS  +  0.05% Tween-20, PBS-T) overnight. The membrane was incubated with polyclonal Rabbit anti-prM (1:1000) (Invitrogen, Cat. #PA5-34966) and purified human anti-Env (fusion loop), 1M7 (2 µg/ml) in 2% BSA + PBS-T solution for 1 h at 37 °C. The primary antigen-antibody complex was detected by incubating the blot with goat antirabbit IgG HRP (1:10000, Jackson-ImmunoLab) and sheep anti-human IgG HRP (1:5000, GE Healthcare) in 5% milk, for 1 h at room temperature. Membranes were developed by Supersignal West Pico PLUS Chemiluminescent Substrate (ThermoFisher). Bands representing E protein and prM were quantitated using imageJ and the ratio of prM/E is reported.

### MiniON real-time sequencing

Viral RNA was isolated from viral stocks using QIAmp Viral RNA Mini Kit (Qiagen #52904), then converted to cDNA using random primers. cDNA was amplified using DENV1-4 Universal primers, Forward primer AAACGCGNGAGAAACCG and Reverse primer TGGAAYTTRTAYTGYTCTGTCC, with 2500 bp coverage across prM and E glycoproteins. Amplicons were pooled using a Zymo DNA Clean and Concentrator kit (Genesee Scientific Cat. 11-303 C). Pooled amplicons were then sequenced using the 1D Ligation Sequencing Kit (Oxford Nanopore Technologies Cat. SQK-LSK109) on the Oxford Nanopore Technologies MinION sequencer following the manufacturer’s recommended protocols. Sequence reads were basecalled within the MinKNOW interface (v.19.05 and greater). Reads were then aligned using the Oxford Nanopore aligner in Geneious Prime (Biomatters). Coverage was greater than 30,000X.

### Illumina total RNA sequencing

Viral genomes were extracted from viral stocks using TRIzol LS, following the manufacturer’s protocol. 100 ng of total RNA was used as input for the Illumina Stranded Total RNA Prep with Ribo-Zero Plus protocol. Libraries were prepared for sequencing on a MiSeq instrument following the manufacturer’s protocol and sequenced on a 150v3 chip with at least 500,000 reads per sample. Reads were aligned to the DENV genome using STAR with the parameters --peOverlapNbasesMin 30 --outSAMtype BAM SortedByCoordinate. BAM files were imported into Geneious Prime and variants with >100 read coverage and >2% frequency were called. Coverage was greater than 20,000X. See Supplementary Fig. 1B for results. Reference codon and percent of change in codon are reported, for example at nucleotide position 1675 the reference codon was aac (aa N) was found in 94.1% of the reads and the changed codon cac (aa H) was found in 5.9% of the reads.

### Antibody-dependent enhancement (ADE)

In vitro ADE experiments were performed as previously described by de Alwis et al.^6^. In brief, serum samples were serially diluted, with starting dilution 1:10 followed by 3-fold serial dilution for a total of 9 dilutions. DENV2 or DENV4 was diluted to achieve an MOI of 1.0 when added to the cells. Equal volumes of DENV2 or DENV4 were then added to the diluted sera, for a final starting dilution of 1:20. The sera plus virus was then incubated at 37 °C for 45 min. K562 cells were then added to the serum-virus complex and incubated for 2 hours at 37 °C. Cells were then washed with twice RPMI, then resuspended in RPMI and incubated at 37 °C for 22 more hours. Cells were then fixed with 4% paraformaldehyde and permeabilized. 1% Normal Mouse Sera was used to block unspecific binding followed by staining with 2H2-Alexa488 (prM Antibody). Flow cytometry was then used to analyze the percent of infected cells.

### Quantification and statistical analysis

Statistical analysis was performed using Prism 7.2 (GraphPad, La Jolla, CA). Variable slope sigmoidal dose-response curves are calculated with top restraint of the calculated asymptote or 100 and bottom restraint of 0. EC_50_ is the concentration of antibody that neutralizes 50% of the virus being tested. Statistical details of experiments can be found in the figure legend.

### Reporting summary

Further information on research design is available in the Nature Portfolio Reporting Summary linked to this article.

## Supplementary information

Supplementary information

Reporting Summary

## Data Availability

Data that support the findings of this study are provided in the Article and Supplementary Information and are available from the corresponding authors upon request. Source data files are provided with this paper. Source data are provided with this paper.

## Source data

Source Data
