# Supplementary material for: A live dengue virus vaccine carrying a chimeric envelope glycoprotein elicits dual DENV2-DENV4 serotype-specific immunity
Source: Nat Commun. 2023 Mar 13;14:1371. doi: 10.1038/s41467-023-36702-x (PMC10009830; doi:10.1038/s41467-023-36702-x)

# A live dengue virus vaccine carrying a chimeric envelope glycoprotein elicits dual DENV2-DENV4 Serotype-Specific Immunity

Ellen Young[1], Boyd Yount[1], Petraleigh Pantoja[2], Sandra Henein [3], Rita M. Meganck[4], Jennifer McBride[1], Jennifer E. Munt[1], Thomas J. Baric[1], Deanna Zhu[1], Trevor Scobey[1], Stephanie Dong[1], Longping V. Tse[7], Melween I. Martinez[5], Armando G. Burgos[5], Rachel L. Graham[1], Laura White[3], Aravinda DeSilva[3], Carlos A. Sariol[2,5,6,7], Ralph S. Baric[1,3,*]

**Affiliations:** [1]Dept. of Epidemiology, Gillings School of Global Public Health, University of North Carolina, Chapel Hill, NC, USA; [2]Unit of Comparative Medicine, University of Puerto Rico-Medical Sciences Campus, San Juan, PR, USA, [3]Dept. of Microbiology and Immunology, School of Medicine, University of North Carolina, Chapel Hill, NC, USA, [4] Dept. of Molecular Microbiology and Immunology, Saint Louis University, St. Louis, MO, USA, [5]Caribbean Primate Research Center, School of Medicine, University of Puerto Rico-Medical Sciences Campus, San Juan, PR, USA, [6]Department of Microbiology and Medical Zoology, University of Puerto Rico-Medical Sciences Campus, San Juan, PR, USA, [7] Department of Internal Medicine, University of Puerto Rico-Medical Sciences Campus, San Juan, PR, USA, * rbaric@email.unc.edu

# Supplementary Figures and Tables

# Supplementary Figure 1

**a**

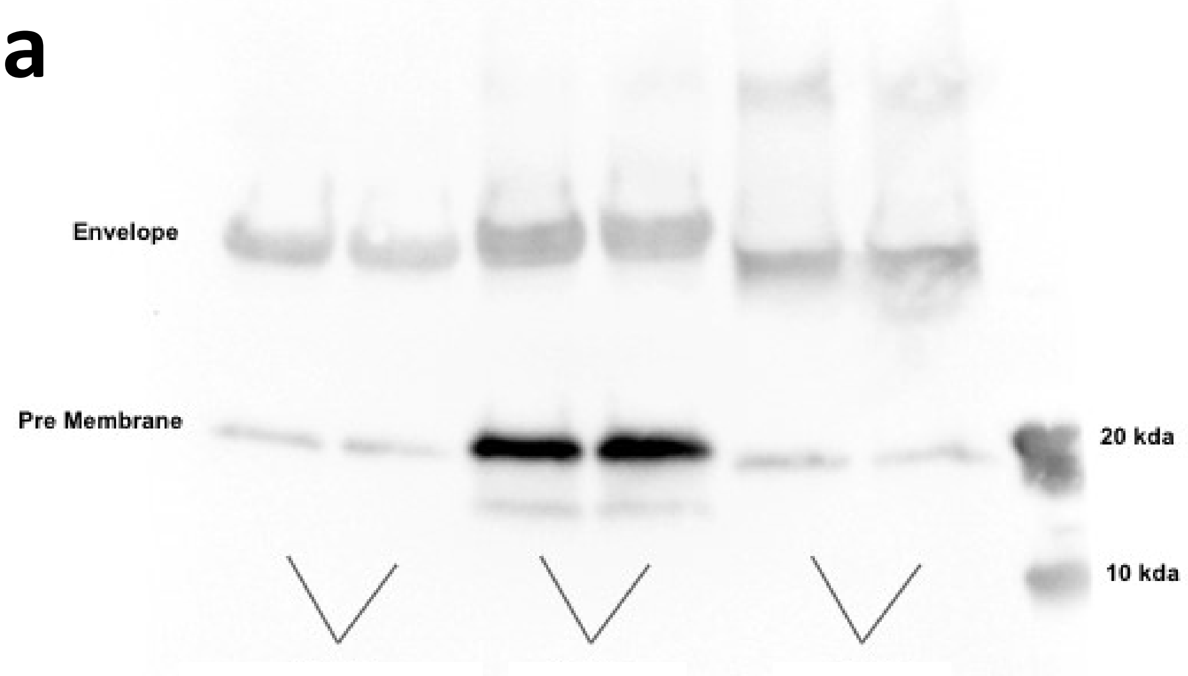


| | DENV2/4EDII | DENV2 | DENV4 | Mol. Wt. Std. |
|---|---|---|---|---|
| prM/E ratio | **0.40** | **2.83** | **0.29** | |

## DENV2/4EDII is more mature and has lower prM content than parental DENV2.

Maturity and prM to E glycoprotein content of DENV2/4EDII is similar to DENV4, as determined by Western Blot. Bands were quantitated using imageJ. Average prM/E ratios are shown.

**b** **DENV2/4 EDII ic deep sequencing by Illumina**

| Position | Coverage | Avg Qual | Ref | Change | Freq | Protein | Protein aa# | Ref Codon | Ref AA | Change Codon | Change AA |
|---|---|---|---|---|---|---|---|---|---|---|---|
| 1625 | 31829 | 32 | A | A -> C | 5.90% | **E** | 230 | aac | N | cac | H |
| 1718 | 29838 | 35 | T | T -> C | 2.90% | **E** | 261 | tac | Y | cac | H |
| 2038 | 30211 | 34 | A | A -> C | 94.80% | **E** | 367 | ata | I | atc | I |
| 2041 | 27218 | 32 | A | A -> G | 7.90% | **E** | 368 | gaa | E | gag | E |
| 2702 | 32500 | 34 | A | A -> G | 4.20% | **NS1** | 94 | aaa | K | gaa | E |
| 5650 | 23435 | 26 | A | A -> G | 2.20% | **NS3** | 376 | aga | R | agg | R |
| 7149 | 38179 | 34 | C | C -> T | 63.40% | **NS4B** | 108 | aca | T | ata | I |
| 7170 | 31368 | 34 | T | T -> C | 11.70% | **NS4B** | 115 | gta | V | gca | A |
| 7190 | 32616 | 30 | G | G -> A | 19.90% | **NS4B** | 122 | ggg | G | agg | R |
| 8472 | 18680 | 34 | A | A -> G | 9.80% | **NS5** | 301 | aaa | K | aga | R |
| 9182 | 9985 | 33 | T | T -> C | 3.70% | **NS5** | 538 | tgg | W | cgg | R |
| 9814 | 13782 | 33 | G | G -> A | 8.10% | **NS5** | 748 | ttg | L | tta | L |
| 10605 | 5427 | 29 | C | C -> T | 3.70% | **3' UTR** | | | | | |
| 10608 | 5299 -> 5390 | 33 | GAA | GAA -> CTT | 2.90% | **3' UTR** | | | | | |

Mutations in blue box are 2 of the 3 TC mutations found in E

**Supplementary Figure 1. DENV2/4EDII is mature and Deep Sequencing quantitates two TC mutations found in the E protein**

a) DENV2/4EDII is more mature and has a lower prM content than parental DENV2 as shown by Western Blot. Duplicate lanes of DENV2/4EDII, DENV2 and DENV4 are shown. Envelope glycoprotein and prM are labeled. Molecular weight standards for 20 kilodaltons and 10 kilodaltons are shown.

b) Deep Sequencing by Illumina of the DENV2/4 EDII chimera identified only two dominant amino acid changes in the E protein with penetrance of greater than 2%, N230H at 5.90% and Y261H at 2.90%. Variants with >100 read coverage and >2% frequency were called.

# Supplementary Figure 2

# Viral proliferation and body temperature for days 1-10 post-infection

A

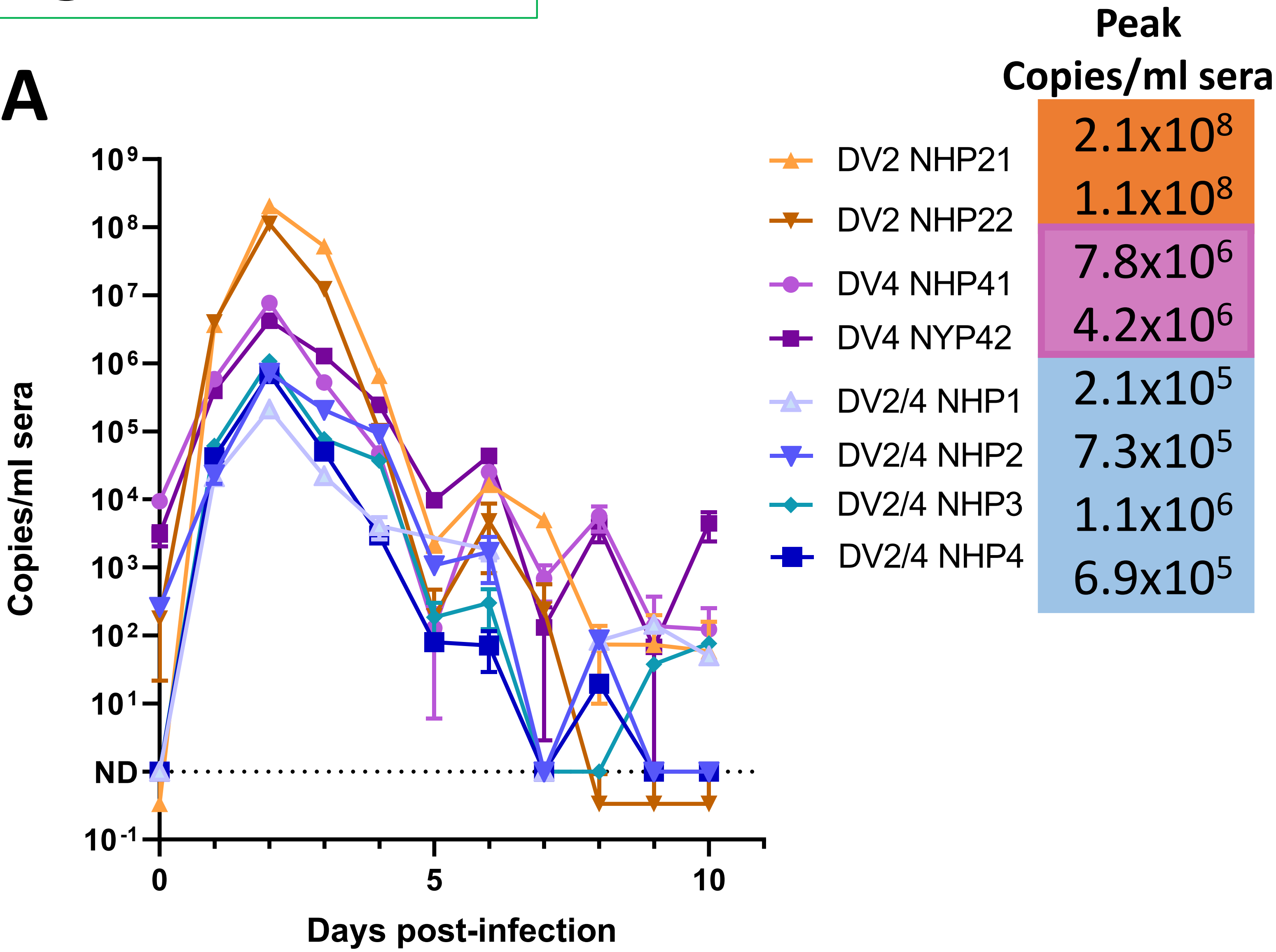


B

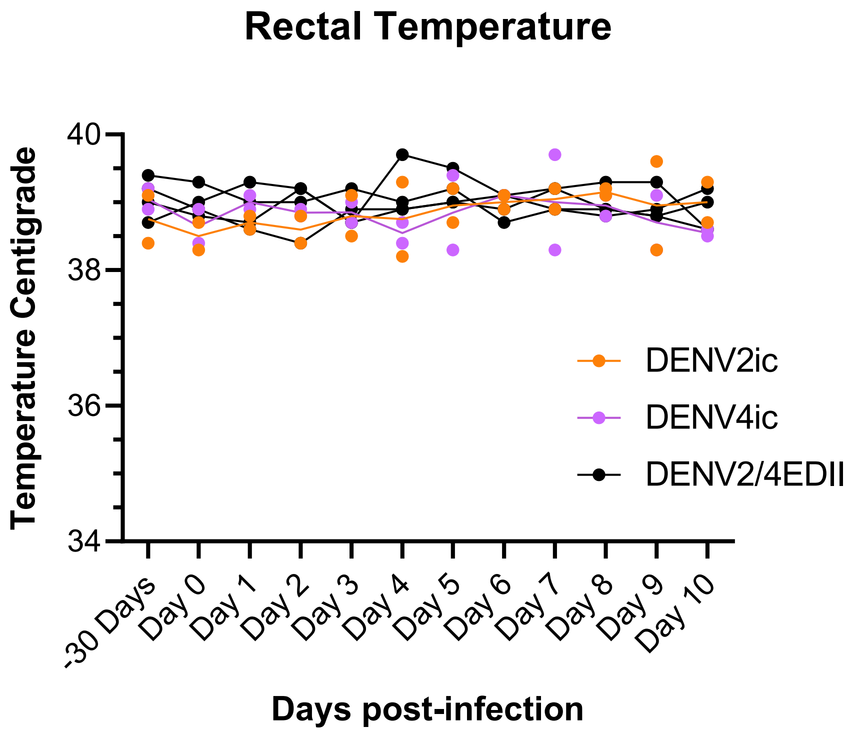

**Supplementary Figure 2. Replication of DENV in NHP and NHP temperature for days 1-10**

a) DENV2/4EDII DENV2 and DENV4 replicated efficiently in Non-Human Primates. Average genome copies/ ml sera, as determined by qrt-pcr, is shown for individual NHP, including two DENV2 (oranges), two DENV4 (purples) and four DENV2/4EDII (blues) inoculated NHP. Values were measured in triplicate. Hashed line represents limit of quantitation (LOQ) for each virus. Mean values with SD are shown. Peak titer for each NHP are show beside key.

b) Macaques were continuously monitored by trained veterinarians. Rectal temperatures were recorded for days 1–10. No significant differences in rectal temperature were observed in most of the animals. Only animal NHP2 reached a temperature discreetly above of the normal internal temperature of rhesus macaques (approximately from 38.5 to 39.3) with peaks of 39.5 $^{o}$C and 39.7 $^{o}$C on days 4 and 5 respectively.

# Supplementary Figure 3 Depletion-ELISA data

a

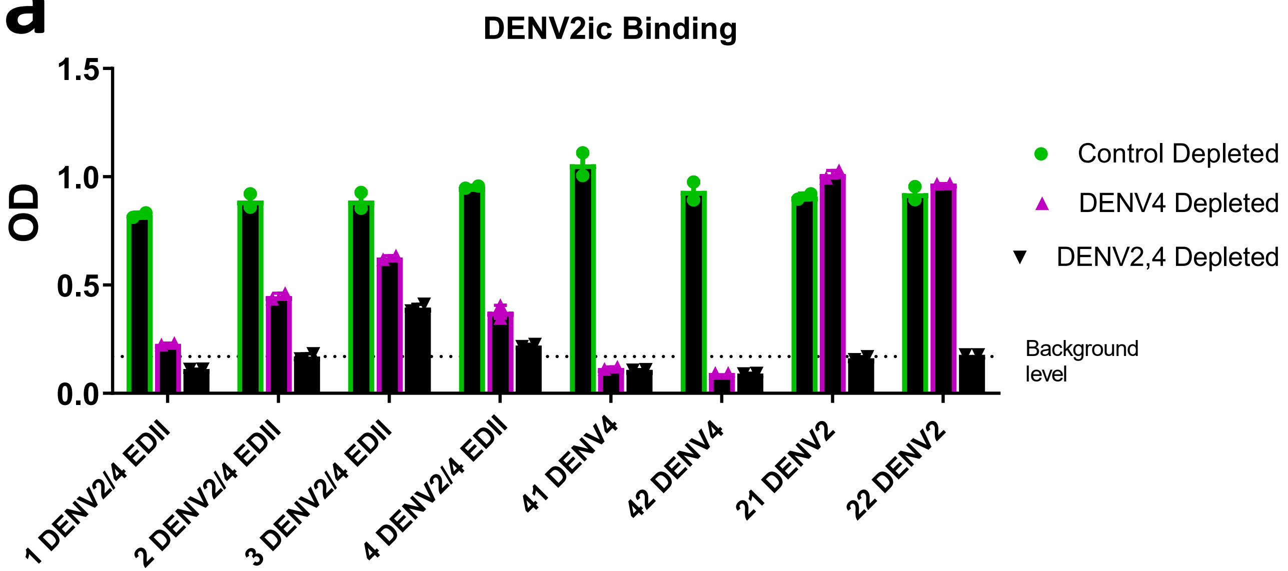


b

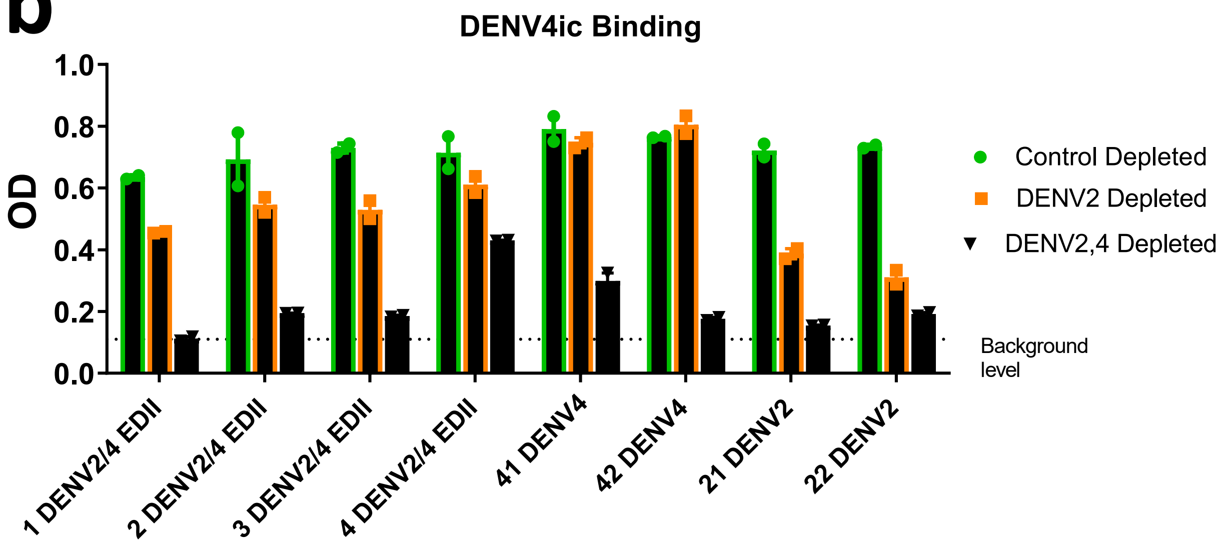


c

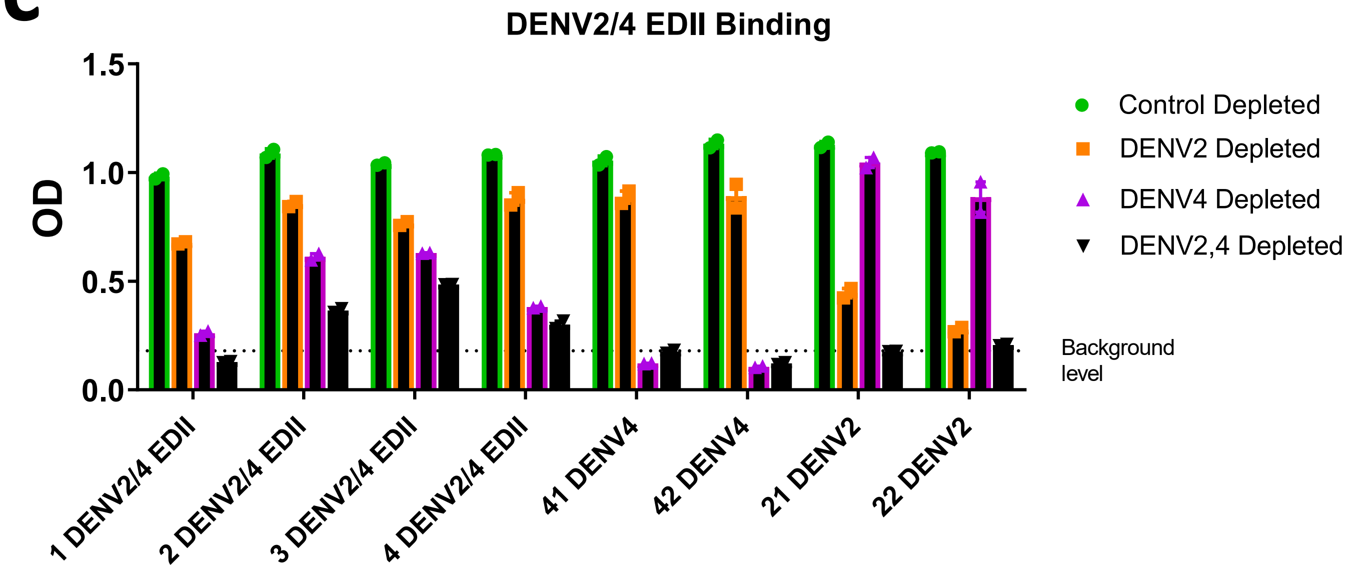

**Supplementary Figure 3. NHP sera depletion confirmed by ELISA.**

a) Depletion of NHP by BSA control (green), DENV4 (purple) or DENV2+DENV4 (black) antigen was confirmed by capture ELISA to whole DENV2 virion. Values measured in duplicate. Bar represents mean +/- SEM.
b) Depletion of sera by BSA control (green), DENV2 (orange) or DENV2+DENV4 (black) antigen was confirmed by capture ELISA to whole DENV4 virion. Values measured in duplicate. Bar represents mean +/- SEM.
c) Depletion of sera by BSA control (green), DENV4 (purple), DENV2 (orange) or DENV2+DENV4 (black) antigen was confirmed by capture ELISA to whole DENV2/4EDII virion. Values measured in duplicate. Bar represents mean +/- SEM.

# Supplementary Figure 4

## Vero-81 FRNT against DENV2

## DENV2/4 EDII

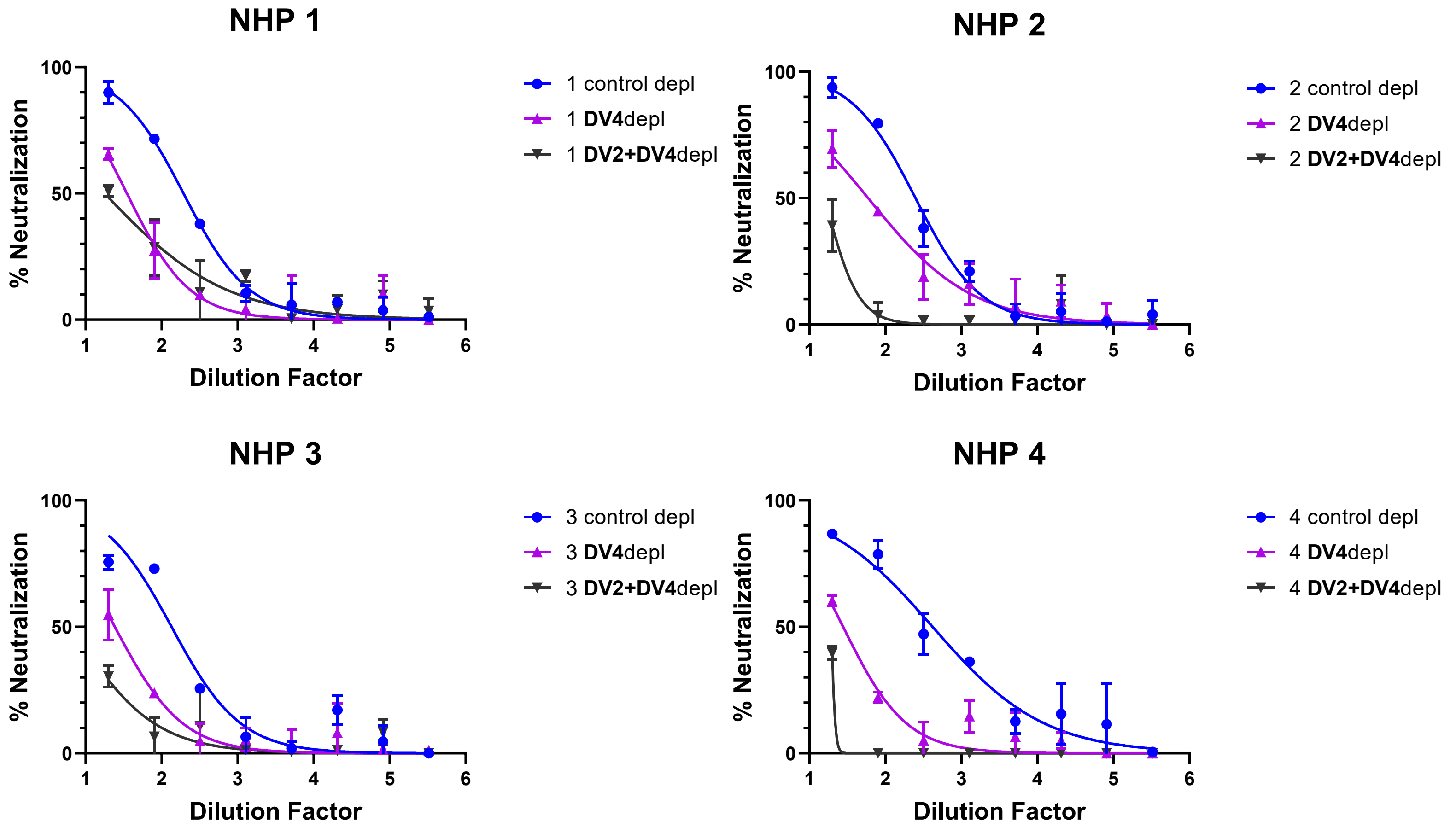


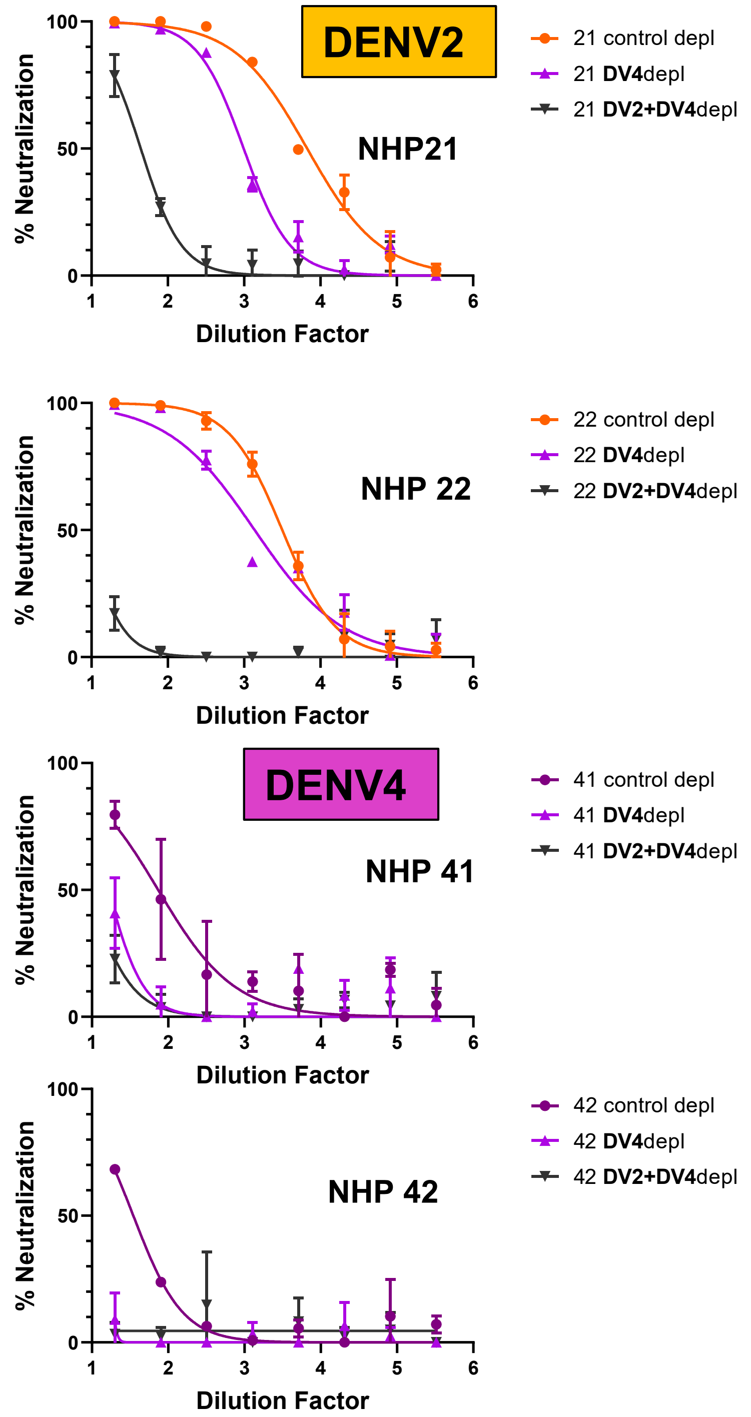

## Supplementary Figure 4. Type Specific Antibodies to DENV2

Neutralization Curves Indicate the Presence of Type-Specific DENV2 antibodies in DENV2/4EDII NHP and DENV2 Sera. The Vero-81 focus reduction neutralization test (FRNT) was used to measure EC50 of depleted sera, Figure 5A. DENV2 neutralization curves for BSA Control depleted, DENV4 depleted and DENV2+DENV4 depleted sera are shown for each individual NHP separately. NHP 1-4 were inoculated with DENV2/4EDII. NHP 21 and 22 were inoculated with DENV2. NHP 41 and 42 were inoculated with DENV4. Each point in the 8-point titration was measured in duplicate. For each point mean +/- SD is shown.

# Supplementary Figure 5

## Vero-81 FRNT against DENV4

## DENV2/4 EDII

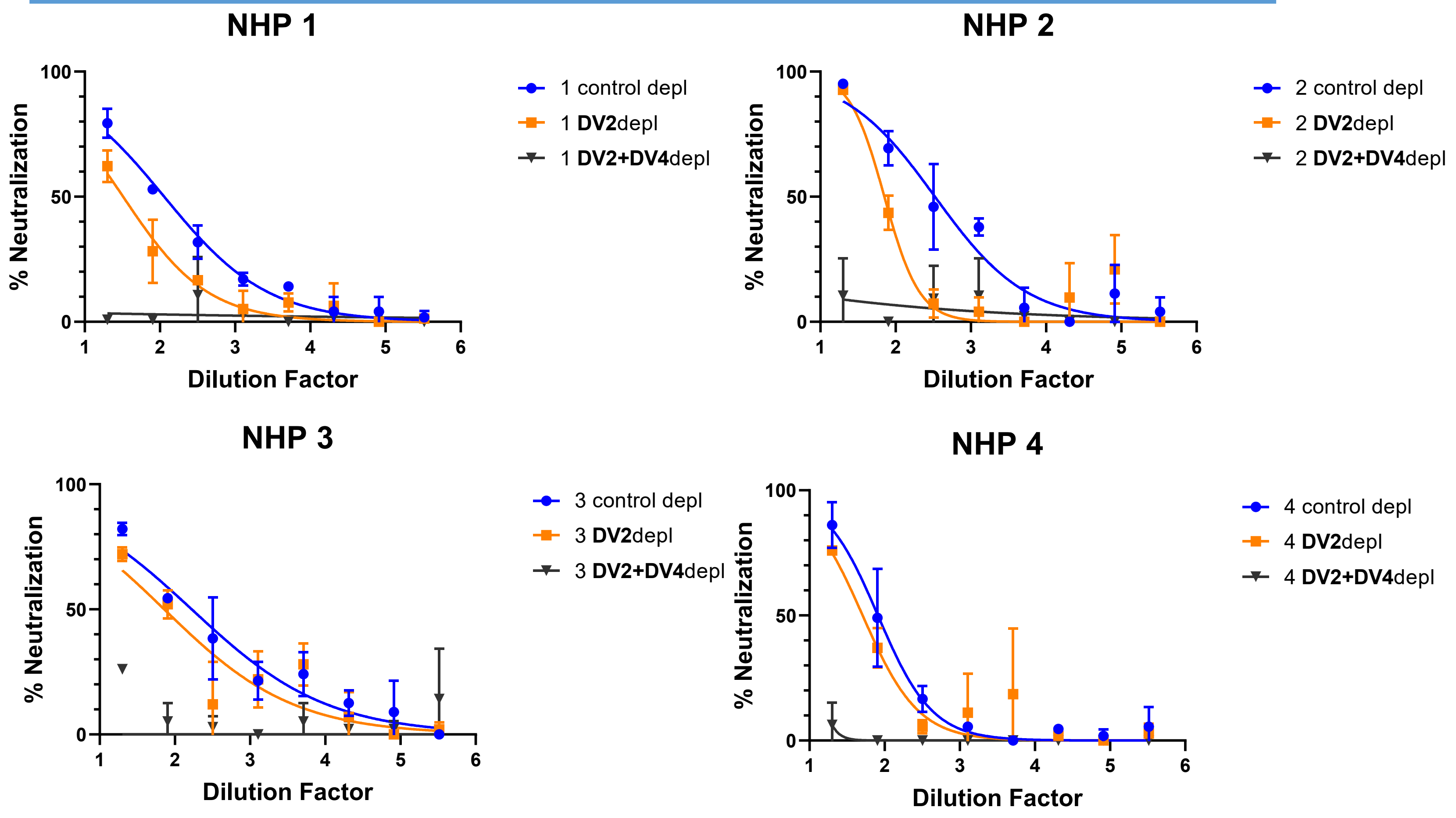


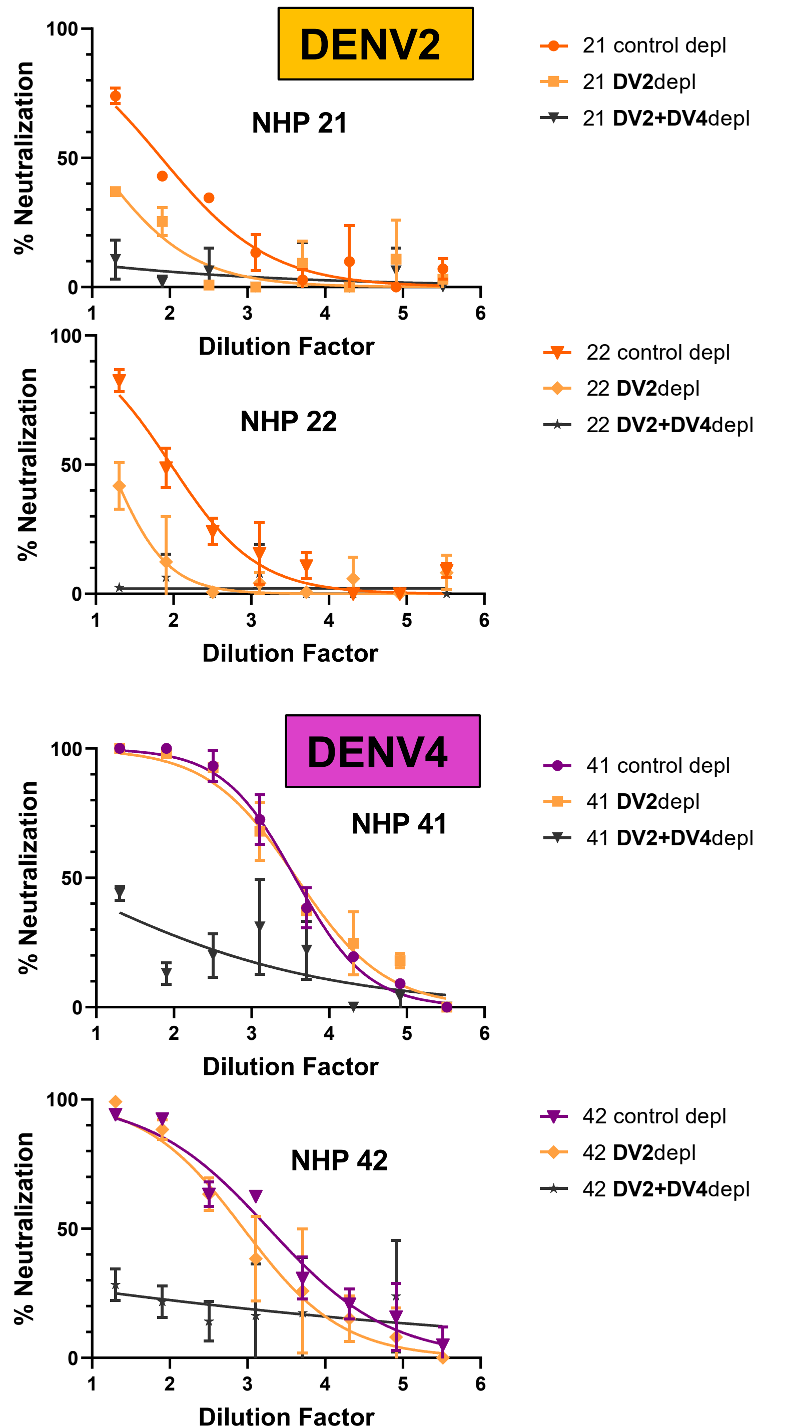

## Supplementary Figure 5. Type Specific Antibodies to DENV4

Neutralization curves indicate the presence of Type-Specific Neutralizing DENV4 antibodies in DENV2/4EDII NHP and DENV4 Sera. Vero-81 focus reduction neutralization test (FRNT) was used to measure EC50 of depleted sera, Figure 5B. DENV4 neutralization curves for BSA Control depleted, DENV2 depleted and DENV2+DENV4 depleted sera are shown for each individual NHP separately. NHP 1-4 were inoculated with DENV2/4EDII. NHP 21 and 22 were inoculated with DENV2. NHP 41 and 42 were inoculated with DENV4. Each point in the 8-point titration was measured in duplicate. For each point mean +/- SD is shown.

# Supplementary Figure 6

## Vero-81 FRNT against DENV2/4 EDII

## DENV2/4 EDII

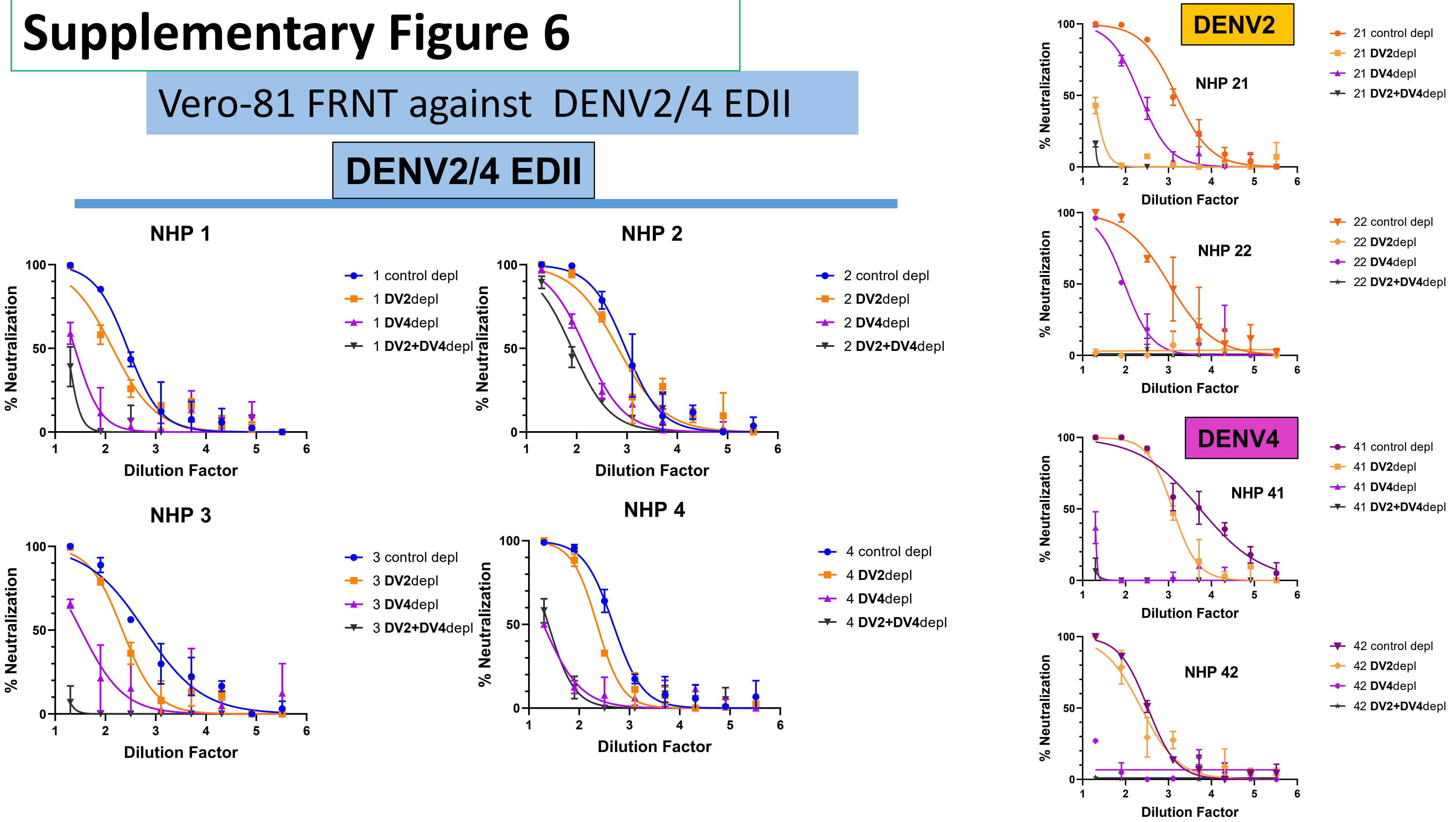

**Supplementary Figure 6. Identification of Type Specific Epitopes in DENV2/4EDII.**

Neutralization curves to DENV2/4EDII identified type-specific DENV2 epitopes in EDI and EDIII, type-specific DENV4 epitopes to EDII and reveal type-specific antibodies that are unique to DENV2/4EDII. Vero-81 focus reduction neutralization test (FRNT) was used to measure EC50 of depleted sera, in Figure 6. DENV2/4EDII neutralization curves for BSA Control depleted, DENV2 depleted, DENV4 depleted and DENV2+DENV4 depleted sera are shown for each individual NHP separately. NHP 1-4 were inoculated with DENV2/4EDII. NHP 21 and 22 were inoculated with DENV2. NHP 41 and 42 were inoculated with DENV4. Each point in the 8-point titration was measured in duplicate. For each point mean +/- SD is shown.

# Supplementary Table 1

## Table of Vero Neut. EC50s

EC50s below detection limit of 20 are listed as 20.
Units are DF (dilution factor)

| | | DV2/4 EDII inocculated | | | | DV2 inocculated | | DV4 onocculated | |
|---|---|---|---|---|---|---|---|---|---|
| **Neut of DV2** | | 1 | 2 | 3 | 4 | 21 | 22 | 41 | 42 |
| | control depl | 194 | 249 | 135 | 419 | 6,699 | 3,160 | 74 | 35 |
| | DV4 depl | 35 | 59 | 24 | 28 | 987 | 1,374 | 20 | 20 |
| | DV2+DV4 depl | 20 | 20 | 20 | 20 | 44 | 20 | 20 | 20 |
| **Neut of DV2 Bkgr subtracted** | | 1 | 2 | 3 | 4 | 21 | 22 | 41 | 42 |
| | control depl | 174 | 229 | 115 | 399 | 6,655 | 3,140 | 54 | 15 |
| | DV4 depl | 15 | 39 | 4 | 8 | 943 | 1,354 | 0 | 0 |
| | DV2+DV4 depl | 0 | 0 | 0 | 0 | 0 | 0 | 0 | 0 |
| **Neut of DV4** | | 1 | 2 | 3 | 4 | 21 | 22 | 41 | 42 |
| | control depl | 114 | 333 | 167 | 81 | 78 | 93 | 3,631 | 1,889 |
| | DV2 depl | 32 | 71 | 72 | 51 | 20 | 20 | 3,777 | 887 |
| | DV2+DV4 depl | 20 | 20 | 20 | 20 | 20 | 20 | 20 | 20 |
| **Neut of DV4 Bkgr subtracted** | | 1 | 2 | 3 | 4 | 21 | 22 | 41 | 42 |
| | control depl | 94 | 313 | 147 | 61 | 58 | 73 | 3,611 | 1,869 |
| | DV2 depl | 12 | 51 | 52 | 31 | 0 | 0 | 3,757 | 867 |
| | DV2+DV4 depl | 0 | 0 | 0 | 0 | 0 | 0 | 0 | 0 |
| **Neut of DV2/4 EDII** | | 1 | 2 | 3 | 4 | 21 | 22 | 41 | 42 |
| | control depl | 278 | 937 | 606 | 478 | 1,507 | 1,043 | 5,458 | 332 |
| | DV2 depl | 139 | 664 | 218 | 227 | 20 | 20 | 1,261 | 233 |
| | DV4 depl | 25 | 150 | 33 | 20 | 216 | 95 | 20 | 20 |
| | DV2+DV4 depl | 20 | 81 | 20 | 24 | 20 | 20 | 20 | 20 |
| **Neut of DV2/4 EDII Bkgr subtracted** | | 1 | 2 | 3 | 4 | 21 | 22 | 41 | 42 |
| | control depl | 258 | 856 | 586 | 454 | 1,487 | 1,023 | 5,438 | 312 |
| | DV2 depl | 119 | 584 | 198 | 203 | 0 | 0 | 1,241 | 213 |
| | DV4 depl | 5 | 69 | 13 | -4 | 196 | 75 | 0 | 0 |
| | DV2+DV4 depl | 0 | 0 | 0 | 0 | 0 | 0 | 0 | 0 |

**Supplementary Table 1. Table of Vero81 Neutralization Assay EC50s**

Depleted Sera Vero-81 FRNT EC50s. Raw and Background Subtracted EC50s of DENV2, DENV4 and DENV2/4EDII Neutralizations are shown. Raw EC50s below detection limit of 20 are listed as 20. Units are dilution factor, DF.

# Supplementary Figure 7a

## DENV2 enhancement in K562 cells

### DENV2 ADE of DV2/4 EDII & DV4 Immune NHP

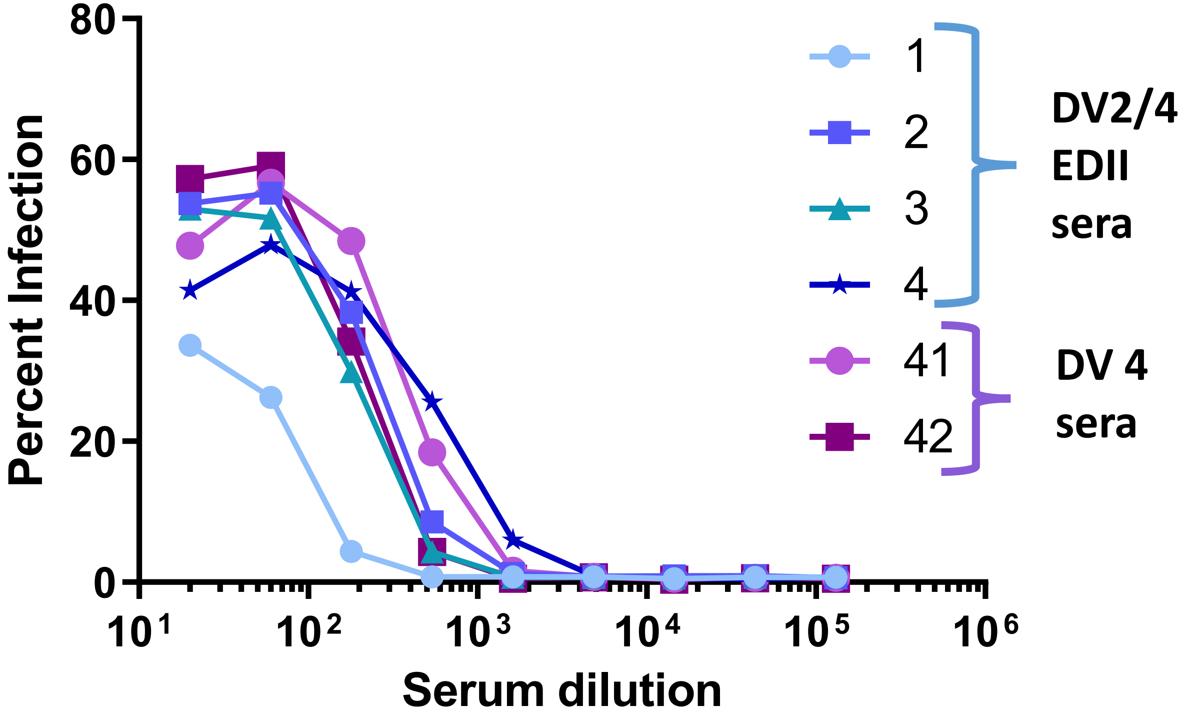


Enhancement of DV2 in K562 cells by both the DV2/4 EDII chimera and DV4 are similar, both in titer and peak percent cells infected.

# Supplementary Figure 7b

## DENV4 enhancementin K562 cells

### DENV4 ADE DV2/4 EDII & DV2 Immune NHP

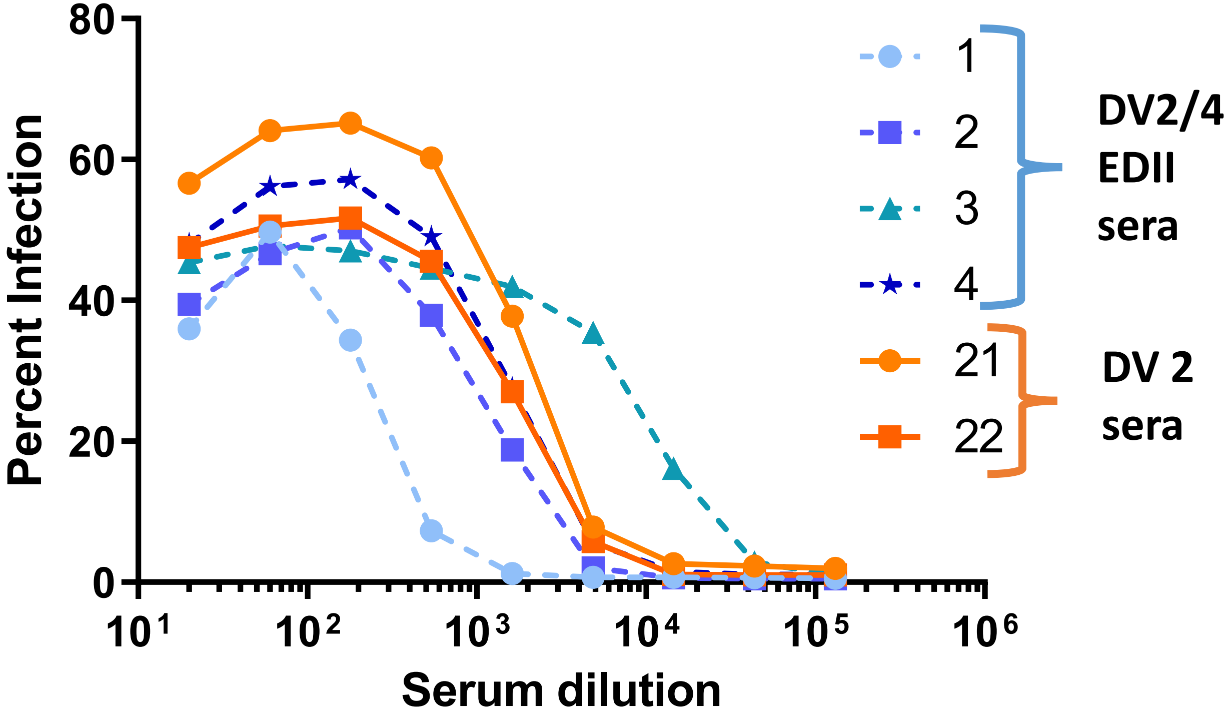


Enhancement of DV4 in K562 cells by both the DV2/4 EDII chimera and DV2 sera are similar, both in titer and peak percent cells infected.

**Supplementary Figure 7. In Vitro Assessment of Enhancement Potential**

a) Enhancement of DV2 in K562 cells by both the DV2/4 EDII chimera and DV4 are similar, both in titer and peak percent cells infected.
b) Enhancement of DV4 in K562 cells by both the DV2/4 EDII chimera and DV2 sera are similar, both in titer and peak percent cells infected.

# Supplementary Figure 8

## Gating Strategy

Histograms show gating strategy used for measurement of percent DENV infected green cells with Guava. Blue arrows show progressive gating. Cells were sequentially gated for singles then cellular morphology. The final histogram shows gate for infected versus uninfected cells. 5000 cells were counted in the final histogram.

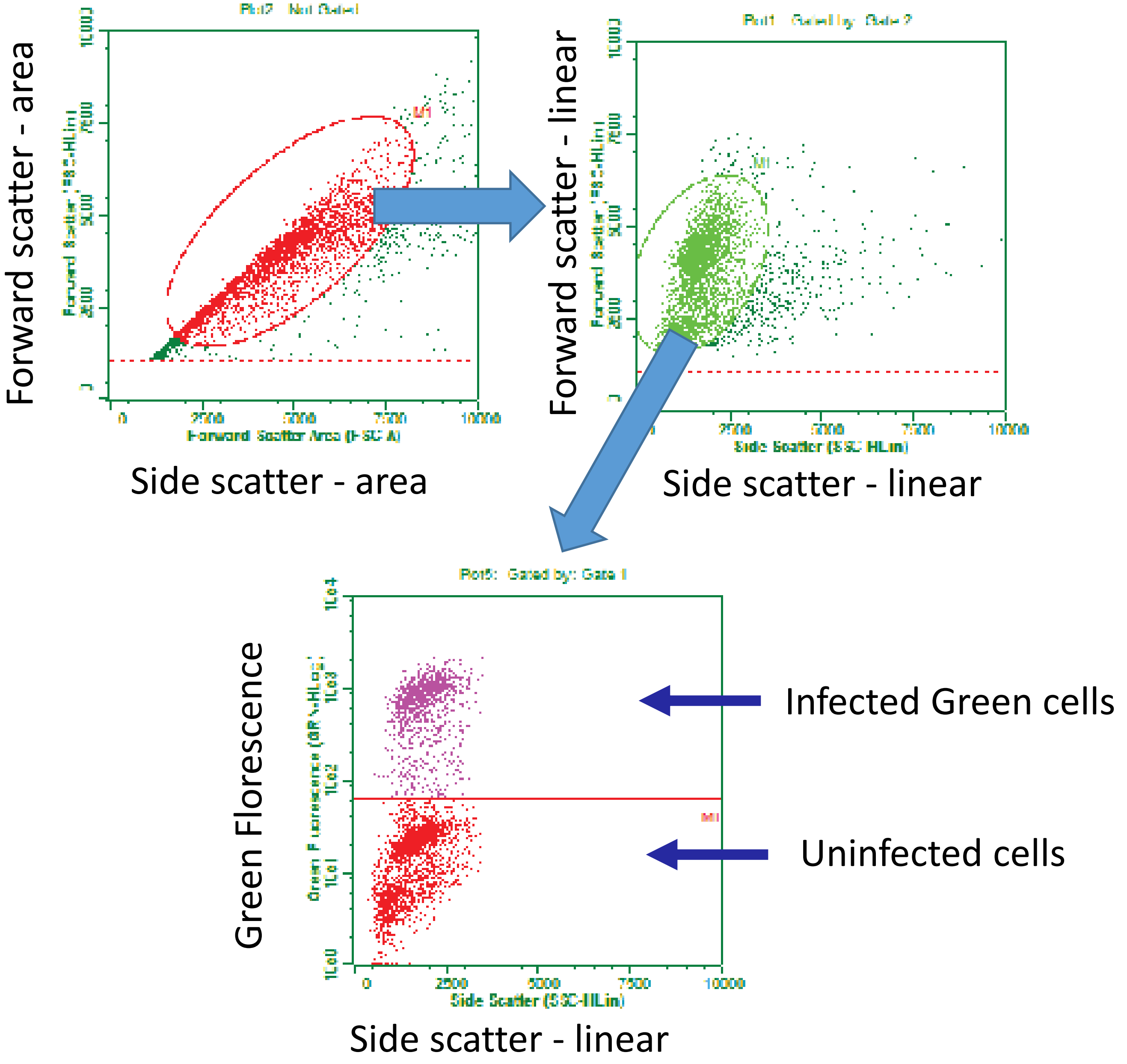

Supplement: Supplementary file 1 — Supplementary information [file 41467_2023_36702_MOESM1_ESM.pdf]
