# Supplementary material for: A live dengue virus vaccine carrying a chimeric envelope glycoprotein elicits dual DENV2-DENV4 serotype-specific immunity
Source: Nat Commun. 2023 Mar 13;14:1371. doi: 10.1038/s41467-023-36702-x (PMC10009830; doi:10.1038/s41467-023-36702-x)
Supplement: Supplementary file 2 — Reporting Summary [file 41467_2023_36702_MOESM2_ESM.pdf]

nature portfolio

Corresponding author(s): Ralph Baric and Ellen Young

Last updated by author(s): Jan 14, 2023

Reporting Summary

Nature Portfolio wishes to improve the reproducibility of the work that we publish. This form provides structure for consistency and transparency in reporting. For further information on Nature Portfolio policies, see our Editorial Policies and the Editorial Policy Checklist.

Statistics

For all statistical analyses, confirm that the following items are present in the figure legend, table legend, main text, or Methods section.

n/a	Confirmed	
☐	☒	The exact sample size (n) for each experimental group/condition, given as a discrete number and unit of measurement
☐	☒	A statement on whether measurements were taken from distinct samples or whether the same sample was measured repeatedly
☒	☐	The statistical test(s) used AND whether they are one- or two-sided<br>*Only common tests should be described solely by name; describe more complex techniques in the Methods section.*
☐	☒	A description of all covariates tested
☐	☒	A description of any assumptions or corrections, such as tests of normality and adjustment for multiple comparisons
☐	☒	A full description of the statistical parameters including central tendency (e.g. means) or other basic estimates (e.g. regression coefficient) AND variation (e.g. standard deviation) or associated estimates of uncertainty (e.g. confidence intervals)
☒	☐	For null hypothesis testing, the test statistic (e.g. F, t, r) with confidence intervals, effect sizes, degrees of freedom and P value noted<br>*Give P values as exact values whenever suitable.*
☒	☐	For Bayesian analysis, information on the choice of priors and Markov chain Monte Carlo settings
☒	☐	For hierarchical and complex designs, identification of the appropriate level for tests and full reporting of outcomes
☒	☐	Estimates of effect sizes (e.g. Cohen's d, Pearson's r), indicating how they were calculated

Our web collection on statistics for biologists contains articles on many of the points above.

Software and code

Policy information about availability of computer code

Data collection	Roche LightCycler 480<br>Immunospot Analyzer software by CTL (Cellular Technology Limited)
Data analysis	Design & Analysis Software 2.5.0 by Applied Biosystems thermofisher<br>GraphPad Prism 7.2 GraphPad Software, Inc. https://www.graphpad.com

For manuscripts utilizing custom algorithms or software that are central to the research but not yet described in published literature, software must be made available to editors and reviewers. We strongly encourage code deposition in a community repository (e.g. GitHub). See the Nature Portfolio guidelines for submitting code & software for further information.

Data

Policy information about availability of data

All manuscripts must include a data availability statement. This statement should provide the following information, where applicable:

- Accession codes, unique identifiers, or web links for publicly available datasets
- A description of any restrictions on data availability
- For clinical datasets or third party data, please ensure that the statement adheres to our policy

Data is available upon request with consent of authors.

Human research participants

Policy information about studies involving human research participants and Sex and Gender in Research.

Reporting on sex and gender	no human subjects
Population characteristics	N/A
Recruitment	N/A
Ethics oversight	N/A

Note that full information on the approval of the study protocol must also be provided in the manuscript.

Field-specific reporting

Please select the one below that is the best fit for your research. If you are not sure, read the appropriate sections before making your selection.

☒ Life sciences ☐ Behavioural & social sciences ☐ Ecological, evolutionary & environmental sciences

For a reference copy of the document with all sections, see nature.com/documents/nr-reporting-summary-flat.pdf

Life sciences study design

All studies must disclose on these points even when the disclosure is negative.

Sample size	Growth curves were performed in triplicate.<br>Antibody/Sera neutralization curves were done using duplicate 8 point dilution curves, so 16 individual measurements are then distilled into one EC50 value for neutralization.<br>Sample size for DENV inoculations were small, DENV2 was 2 macaques, DENV4 was 2 macaques and DENV2/4EDII was 4 macaques. It is noted in the manuscript that the number of macaques was small and this limits our conclusions.
Data exclusions	There were no data exclusions.
Replication	Similar results were found in all reproduced experiments not found in this manuscript. So we consider all results to be reproducible.
Randomization	Randomization of growth curves and and antibody neutralization measurements would not be appropriate for these small studies.<br>Young rhesus macaques (2.5-4 years of age) seronegative for DENV and ZIKV were housed in the CPRC facilities, University of Puerto Rico, San Juan, Puerto Rico. All macaques were male. The eight male macaques selected for this study were randomly placed in group to be inoculated with DENV2, DENV4 or DENV2/4EDII.
Blinding	Samples were not blinded. It is not common practice in this field of study to blind the samples used in a study of this size. This is not a clinical study.

Reporting for specific materials, systems and methods

We require information from authors about some types of materials, experimental systems and methods used in many studies. Here, indicate whether each material, system or method listed is relevant to your study. If you are not sure if a list item applies to your research, read the appropriate section before selecting a response.

Materials & experimental systems

n/a	Involved in the study	
☐	☒	Antibodies
☐	☒	Eukaryotic cell lines
☒	☐	Palaeontology and archaeology
☐	☒	Animals and other organisms
☒	☐	Clinical data
☒	☐	Dual use research of concern

Methods

n/a	Involved in the study	
☒	☐	ChIP-seq
☐	☒	Flow cytometry
☒	☐	MRI-based neuroimaging

Antibodies

Antibodies used	EDE1-C8 (plasmid-produced Ig),EDE2-B7(plasmid-produced Ig), DENV-2D22 (hybridoma-produced Ig), DENV-3F9 (hybridoma-produced Ig), DENV-126 (hybridoma-produced Ig), DENV-131 (hybridoma-produced Ig), DENV-4G2(hybridoma-produced Ig),DENV-2H2(hybridoma-produced Ig),Goat anti-Human IgG (Fc)-AP
Validation	All DENV specific hybridoma-produced Ig were tested against serotypes 1-4 of DENV to assure specificity. They were also tested against appropriate DENV chimeras to assure intended accruacy of the epitope.

Eukaryotic cell lines

Policy information about cell lines and Sex and Gender in Research

Cell line source(s)	C6/36 cells , source ATCC Cat# CRL-1660<br>Vero-81 cells , source ATCC Cat# CCL-81<br>K562 cells , source ATCC Cat# CCL-243<br>U937-DC-SIGN cells, source ATCC Cat# CRL-3253
Authentication	Stocks from ATCC were immediately frozen down to serve as primary stocks. Cells were monitored for morphology and reactivity.
Mycoplasma contamination	All cell lines were tested and tested negative for mycoplasma contamination.<br>Testing was done through the UNC Lineberger Tissue Culture Facility which partners with Genetica Cell Line Services (part of LabCorp) to offer the most stringent qPCR-based Mycoplasma contamination testing
Commonly misidentified lines<br>(See ICLAC register)	No commonly misidentified cell lines were used in the study.

Animals and other research organisms

Policy information about studies involving animals; ARRIVE guidelines recommended for reporting animal research, and Sex and Gender in Research

Laboratory animals	Young rhesus macaques (2.5-4 years of age) seronegative for DENV and ZIKV were housed in the CPRC facilities, University of Puerto Rico, San Juan, Puerto Rico
Wild animals	No wild animals were used in the study.
Reporting on sex	All Macaques were male.
Field-collected samples	No field collected samples were used in the study.
Ethics oversight	ll procedures were reviewed and approved by the Institute’s Animal Care and Use Committee at Medical Sciences Campus, University of Puerto Rico (IACUC-UPR-MSC) and performed in a facility accredited by the Association for Assessment and Accreditation of Laboratory Animal Care (AAALAC) (Animal Welfare Assurance number A3421; protocol number, 7890116). Procedures involving all study animals were approved by the Medical Sciences Campus, UPR IACUC and were conducted in accordance with USDA Animal Welfare Regulations, the Guide for the Care and use of Laboratory Animals and institutional policies. In addition, steps were taken to lighten sufferings in accordance with the recommendations of the Guide for the Care and use of Laboratory Animals (8th edition), Animal Welfare Act and the Public Health Service (PHS) Policy on Humane Care and Use of Laboratory Animals.

Note that full information on the approval of the study protocol must also be provided in the manuscript.

Flow Cytometry

Plots

Confirm that:

- [x] The axis labels state the marker and fluorochrome used (e.g. CD4-FITC).
- [x] The axis scales are clearly visible. Include numbers along axes only for bottom left plot of group (a 'group' is an analysis of identical markers).
- [x] All plots are contour plots with outliers or pseudocolor plots.
- [x] A numerical value for number of cells or percentage (with statistics) is provided.

Methodology

Sample preparation	U937-DC-SIGN cells were fixed, permiablized and stained with a single monoclonal antibody, DENV-prM-2H2-Alexa488.
Instrument	Guava

Software	GuavaSoft ExpressPro 2.2.3
Cell population abundance	No less than 5000 cells were counted in the final gated histogram that measured the percent of cells with green fluorescence in the sample.
Gating strategy	Cells were gated on Forward Scatter area vs Side Scatter area to select singlets. Singlet events were sent to Forward Scatter Linear vs Side Scatter Linear to select for correct cell size and morphology. Cells passing both gates were assessed for percent green.

☒ Tick this box to confirm that a figure exemplifying the gating strategy is provided in the Supplementary Information.
